# Helicobacter pylori Evolution: Lineage- Specific Adaptations in Homologs of Eukaryotic Sel1-Like Genes

**DOI:** 10.1371/journal.pcbi.0030151

**Published:** 2007-08-10

**Authors:** Masako Ogura, J. Christian Perez, Peer R. E Mittl, Hae-Kyung Lee, Geidrius Dailide, Shumin Tan, Yoshiyuki Ito, Ousman Secka, Daiva Dailidiene, Kalyani Putty, Douglas E Berg, Awdhesh Kalia

**Affiliations:** 1 Department of Molecular Microbiology, Washington University School of Medicine, St. Louis, Missouri, United States of America; 2 Department of Biochemistry, University of Zürich, Switzerland; 3 Second Department of Internal Medicine, Fukui Medical School, Fukui, Japan; 4 Program on Disease Evolution, University of Louisville, Louisville, Kentucky, United States of America; 5 Department of Biology, University of Louisville, Louisville, Kentucky, United States of America; 6 Department of Genetics, Washington University School of Medicine, St. Louis, Missouri, United States of America; 7 Department of Medicine, Washington University School of Medicine, St. Louis, Missouri, United States of America; 8 Department of Microbiology and Immunology, University of Louisville, Louisville, Kentucky, United States of America; University of California Davis, United States of America

## Abstract

Geographic partitioning is postulated to foster divergence of Helicobacter pylori populations as an adaptive response to local differences in predominant host physiology. *H. pylori's* ability to establish persistent infection despite host inflammatory responses likely involves active management of host defenses using bacterial proteins that may themselves be targets for adaptive evolution. Sequenced H. pylori genomes encode a family of eight or nine secreted proteins containing repeat motifs that are characteristic of the eukaryotic Sel1 regulatory protein, whereas the related *Campylobacter* and *Wolinella* genomes each contain only one or two such “Sel1-like repeat” (SLR) genes (“*slr* genes”). Signatures of positive selection (ratio of nonsynonymous to synonymous mutations, *d_N_/d_S_* = ω > 1) were evident in the evolutionary history of *H. pylori slr* gene family expansion. Sequence analysis of six of these *slr* genes (*hp0160, hp0211, hp0235, hp0519, hp0628,* and *hp1117*) from representative East Asian, European, and African H. pylori strains revealed that all but *hp0628* had undergone positive selection, with different amino acids often selected in different regions. Most striking was a divergence of Japanese and Korean alleles *of hp0519,* with Japanese alleles having undergone particularly strong positive selection (ω_J_ > 25), whereas alleles of other genes from these populations were intermingled. Homology-based structural modeling localized most residues under positive selection to SLR protein surfaces. Rapid evolution of certain *slr* genes in specific H. pylori lineages suggests a model of adaptive change driven by selection for fine-tuning of host responses, and facilitated by geographic isolation. Characterization of such local adaptations should help elucidate how H. pylori manages persistent infection, and potentially lead to interventions tailored to diverse human populations.

## Author Summary

Helicobacter pylori is a genetically diverse bacterial species that infects billions of people worldwide, typically for decades. Long-term infection is a major risk factor for stomach ulcers and cancer, although most infections are benign, and the risks of various disease outcomes vary markedly among human populations. Analyses of housekeeping genes, whose encoded proteins perform normal cellular metabolic functions, had established that H. pylori strains from different geographic regions differed in their DNA sequences. Here, we analyzed the *H. pylori slr* multigene family that encodes up to nine secreted proteins (called SLR proteins) quite similar to the human protein Sel1. We showed that most members of the *H. pylori slr* gene family evolved significantly more rapidly than normal housekeeping genes. Different amino acids were selected in different H. pylori lineages, often on exposed surfaces of SLR proteins where they were potentially positioned to interact with host components. We propose that these amino acid differences affect the SLR protein function, likely contributing to *H. pylori's* adaptation to local differences in human stomach physiology. Further characterization of H. pylori proteins with lineage-specific differences in amino acids should improve understanding of geographic differences in H. pylori–host interactions and human disease, and of the interplay between different evolutionary forces in natural populations of any species.

## Introduction

Helicobacter pylori chronically infects billions of people worldwide, typically for decades. Most H. pylori reside on gastric epithelial cell surfaces and in the overlying mucin layer, a tissue that turns over rapidly, is infiltrated by inflammatory cells after infection, and is buffeted by gastric acidity on its luminal side. The gastric mucosa is hostile to most bacterial species, and constitutes an unstable niche to which only the *Helicobacters* among bacterial taxa have become well adapted, in part perhaps through effective management of host responses to infection [1–4].

Great genetic diversity, geographic differences in predominant genotypes, and rapid evolvability are hallmarks of H. pylori populations [5–9]. Independent isolates generally differ by some 2% or more in DNA sequence of any metabolic (housekeeping) gene, with most such differences being synonymous (protein sequences unchanged). Phylogenetic analyses of H. pylori housekeeping gene sequences revealed differences in predominant genotypes between East Asian, European, and African populations that are far greater than those seen with most other pathogens. Such patterns reflect a combination of mutation, recombination, selection to retain gene function, and random genetic drift, which itself likely stems from *H. pylori's* highly localized (preferentially intrafamilial; nonepidemic) mode of transmission, and a resulting relative lack of H. pylori gene flow between well-separated human populations [6,7,10–12]. Correlated with this geographic partitioning of H. pylori populations are striking differences in predominant clinical consequences of infection. To illustrate, duodenal ulcer, which is typically associated with excess gastric acidity (hyperchlorhydria), is far more common in India than in Japan, whereas gastric cancer, which is typically associated with hypochlorhydria, is far more common in Japan than in India [13,14]. The near universality of H. pylori infection until very recently, the extraordinary chronicity of infection, and geographic differences among H. pylori populations all have contributed to an idea that H. pylori may have co-evolved with its human hosts [15]. Geographically isolated populations are also more likely to adapt to differences in local environment [16,17]. Human genetic or physiological traits that diverged during our evolution, that differ geographically, and that are important to H. pylori could have selected for adaptive changes in cognate H. pylori genes.

The virulence-associated *cagA*, and *vacA* genes provide examples in which evolutionary dynamics are likely to have been shaped by local differences in host physiology. CagA and VacA proteins each enter target cells and affect several normal cellular signal transduction pathways, with strengths and specificities that vary geographically. For example, East Asian and Western type CagA proteins differ most in sequences of domains responsible for phosphorylation and in resulting interactions with host SHP-2 phosphatase, an intracellular regulator of various cell proliferative, morphogenetic, and motility signaling pathways [18,19]. Similarly, highly active “*s1,m1*”–type alleles of the *vacA* toxin gene predominate in Japan and Korea, whereas nontoxigenic *s2,m2*–type alleles are common in the West [20,21], and a recombinant *s1,m2* form predominates in coastal China [22]; the “m” region of VacA determines the cell type specificity of toxin action. We suggest that these geographic differences reflect types of selection pressures that predominate(d) in the various human populations, either currently or in centuries past, superimposed on the random genetic drift that figures so importantly in geographic partitioning of housekeeping genes; and that such patterns may be common among genes whose products interact with host components. Furthermore, the extraordinary chronicity of H. pylori infection suggests a possible need for effective management and potentially even exploitation of host responses. For example, although inflammatory responses help protect potential hosts against casual pathogen encounters, H. pylori is thought also to use metabolites leached from inflammation-damaged host tissues for its nutrition [23]. In addition, many strains use host sialylated glycolipids, synthesized during the inflammatory response, as receptors for adherence [24]. In this framework, much of *H. pylori*-induced gastric pathology might reflect how host signaling pathways are modulated by contact with the bacterium or its secreted products.

**Figure 1:**
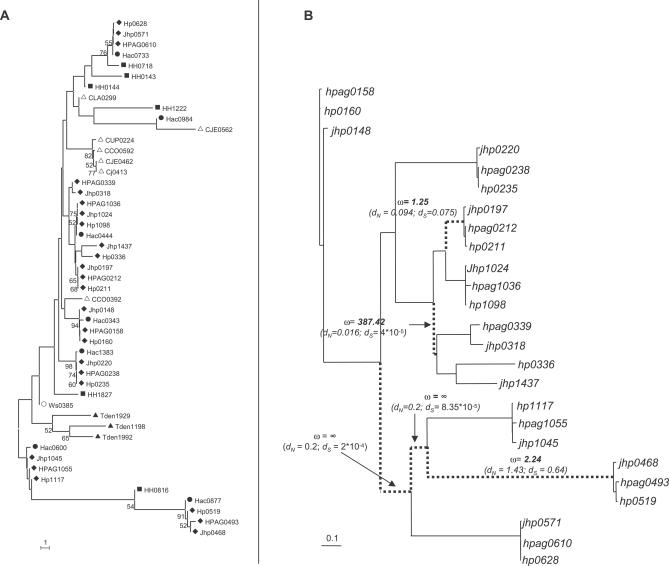
SLR Gene Family Evolution (A) Phylogenetic relationships of *slr* gene family homologs in ɛ-proteobacteria: *Helicobacter* genome-specific expansion. *slr* homologs were identified in ten fully sequenced genomes of closely related ɛ-proteobacterial genera using the *COG* database. A multiple sequence alignment was generated by aligning SLR protein sequences to crystal structures of *H. pylori* SLR proteins Hp0336 [57] and Hp1098 [58] using *EXPRESSO* (http://igs-server.cnrs-mrs.fr/Tcoffee/tcoffee_cgi/index.cgi), which aligns pairs of structures with SAP while sequence–structure pairs are aligned with *FUGUE*. The resulting collection of pairwise alignments was combined into a multiple sequence alignment with the *T-COFFEE* algorithm. A neighbor-joining tree was reconstructed using the Jones-Tyler-Thornton distances, and rate heterogeneity among sites was modeled with a discrete Γ distribution with shape parameter α = 0.5, as implemented in MEGA version 3.1 (http://www.megasoftware.net). Bootstrap values ≥50 were considered significant and are indicated on individual branches. Scale indicates one amino acid substitution per site. Strain abbreviations used and genomes included are as follows: Hp, Jhp and HPAG, for H. pylori genomes from strains 26695 [39], J99 [40], and HPAG1 [41], respectively; Hac, H. acinonychis strain Sheeba [42]; Hh, H. hepaticus ATCC 5149 [44]; CLA, C. lari RM2100 (Genbank AAFK00000000); CUP, C. upsaliensis RM3195 (Genbank AAFJ00000000); CCO, C. coli RM2228 (Genbank AAFL00000000); Cj, *C. jejuni subsp. jejuni* NCTC 11168 [65]; CJE, C. jejuni RM1221 [66]; Ws, Wolinella succinogenes DSM1740 [45]; and Tden, Thiomicrospora denitrificans ATCC 33889 (Genbank CP000153). (B) Multiple episodes of positive selection in *H. pylori slr* gene family expansion. A structure-based alignment of H. pylori SLR proteins from strains 26695, J99, and HPAG1 was derived using *EXPRESSO* as described above; a corresponding *slr* gene family sequence alignment was derived manually. An initial ML tree, used as input for selection analysis, was generated assuming the TrN + I + Γ model of sequence evolution (parameter estimates are available from authors on request). Phylogeny shown above was estimated under the FR model implemented in PAML version 3.14. Branches that experienced positive selection during their evolution are indicated by dotted lines, and *d_N_*, *d_S_*, and *ω* values are indicated. An ω ratio = ∞ indicates branches that only accumulated nonsynonymous mutations during their divergence. *d_N_*, *d_S_*, and *ω* values for all other branches in the phylogeny are shown in Table S4. Scale indicates number of substitutions per codon.

Sequenced H. pylori genomes contain a gene-family whose encoded proteins are likely secreted, and contain two or more copies of a degenerate 34–36 amino acid repeat motif that is characteristic of eukaryotic “Sel1” proteins, which themselves help regulate diverse signal transduction pathways [25]. The proteins bearing this repeat are typically built of several consecutive α/α motifs, the antiparallel α-helices of the motifs being connected by a short loop [26]. Five of the nine members of this “SLR” (for Sel1-like repeat) protein family are rich in cysteine residues and had been designated as “*Helicobacter* cysteine rich proteins” (Hcp) [27,28]; The α-helices of each SLR repeat are bridged by a disulfide bond, which is a unique feature of Hcps [26]. Although the in vivo function of H. pylori SLR proteins is not known, some Hcps bind β-lactam compounds [27,29], which suggests possible interactions with immunomodulatory peptidoglycan fragments, that could affect the innate immune response [30]. High antibody titres against four SLR proteins [Hp0211 (HcpA), Hp0235 (HcpE), Hp0336 (HcpB), and Hp1098 (HcpC)] were found in H. pylori–infected people [28], indicating in vivo expression and immune recognition. Furthermore, recombinant HcpA elicited IL12-dependent IFN-γ secretion in a naïve mouse splenocyte model [30], and HP1117 elicited protective antibodies during mouse infection [31]. Only one or two *slr* homologs are found in members of the closely related *Campylobacter* and *Wolinella* genera, whereas strains of H. acinonychis (from big cats) and of the nongastric mouse pathogen H. hepaticus (implicated in liver cancer) contain seven and six *slr* homologs, respectively (Figure 1A). It is appealing to imagine that this expanded family of secreted proteins affects bacterial–host interactions during chronic *Helicobacter* infection of mucosal tissues.

Here, we posited that if geographically isolated H. pylori populations had adapted to local differences in host physiology caused by factors such as host nutrition, genotype, or infection by other pathogens that in turn impacted on responses to H. pylori, these adaptations would leave an imprint of natural selection on the affected genes, superimposed on *H. pylori's* overall population genetic structure. For protein-coding genes, selection pressures and adaptive evolution can be detected and examined by comparing rates of fixation of synonymous (silent; *d_S_*) versus nonsynonymous (amino acid altering; *d_N_*) mutations in the population [32,33]. The ratio *d_N_*/*d_S_* (= ω) indicates whether amino acid change is unaffected, inhibited, or promoted by natural selection. Considering that most synonymous substitutions have little if any effect on fitness, *d_S_* is often equated to the rate of neutral nucleotide substitution. Under neutral evolution, one would expect *d_N_* and *d_S_* to be equal (ω = 1), and functionally critical genes (e.g., housekeeping genes, responsible for intracellular metabolic functions) to show very low *d_N_* (ω < 1) [32]. In certain genes, however, nonsynonymous substitutions are in excess (ω > 1) because changes in their encoded proteins are advantageous and thus have been selected in particular environmental contexts. This is often termed positive selection (sometimes also called diversifying or Darwinian selection). Many such substitutions are likely to have been selected specifically to change the activity or structure of encoded proteins [34]. Since differences in local conditions (e.g., host features) can lead to geographic differences in patterns of selection [16], evolutionary rates of amino acid substitutions are likely to vary among H. pylori lineages; and an elevated *d_N_* (ω >>1) in any specific lineage would indicate adaptive evolution. Such adaptive evolution tends to be episodic, in that it operates sporadically, and affects only a few amino acid positions in the protein [34]. Consequently, methods that estimate average *d_N_* and *d_S_* (summed over all codons and all lineages) often fail to detect adaptive evolution [33]. Here, we applied codon-based models of sequence evolution in conjunction with maximum-likelihood (ML) computational methods [35,36] that are particularly useful for detecting adaptive changes at specific sites in proteins, to study the evolutionary dynamics of the *H. pylori slr* gene family. We found that most *slr* family members had experienced positive selection, and accumulated adaptive mutations in specific H. pylori lineages, preferentially affecting specific surface-exposed sites in encoded proteins. These outcomes suggested selection for management and fine-tuning of host responses during chronic infection. Our results illustrate the utility of population-based phylogenetic strategies for identifying human population-specific adaptive determinants of *H. pylori.*

## Results/Discussion

### *H. pylori slr* Gene Family

This study began with subtractive hybridization (as in [37,38]) to find genes or alleles that differed markedly between representative Japanese versus Western strains. One recovered clone contained a fragment of gene *hp0519.* Subsequent sequencing of *hp0519* alleles from representative strains identified two in-frame deletions: a 24-bp segment that was absent from the Japanese strain (Δ24) but present in US reference strain J99 (*nt 133–156*), and a 15-bp segment in the Japanese strain that was absent from J99 (Δ15) (*nt 640–654*). PCR tests indicated that 70 of 87 Japanese strains carried Δ24 15+ type alleles, and only 14 carried the reciprocal 24+ Δ15 type allele, whereas 45 of 47 Spanish strains tested carried 24+ Δ15, the allele type that was uncommon in Japanese strains. Remarkably, 24 of 28 Korean strains tested also carried 24+ Δ15 type alleles, not the Δ24 15+ type that predominated in Japan. This difference in *hp0519* pattern seemed extraordinary because Japanese and Korean strains were closely related in sequences of all other *slr* genes tested (see below); they were also closely related in sequences of housekeeping genes (Figure S2B; and Dailide and Berg, unpublished), which should be subject only to purifying selection to maintain function within the bacterial cell. Such relatedness was expected given the proximity of Japan and Korea and the shared history of their peoples.

Inspection of the Hp0519 protein sequences using the SignalP (http://www.cbs.dtu.dk/services/SignalP) and SMART (simple modular research tool) (http://smart.embl-heidelberg.de/) programs identified an N-terminal signal sequence and three SLRs with 40% sequence similarity to the human Sel1L protein (Tables S1 and S2; Figure S1A). Hp0519 belongs to the cluster of orthologous group (COG0790), which contains eight other family members in the genome of H. pylori strain 26695 ([39], and seven in the genomes of strains J99 [40] and HPAG1[41]) (http://www.ncbi.nlm.nih.gov/COG) (Figure 1A). Each COG0790 family member is predicted to encode a secreted protein with two or more SLRs (Table S2). Seven of these *slr* genes are present in each of the three sequenced genomes—*hp0160 (jhp0148/hpag0158), hp0211 (jhp0197/hpag0212), hp0235 (jhp0220/hpag0238), hp0519 (jhp0468/hpag0493), hp0628 (jhp0571/hpag0610), hp1098 (jhp1024/hpag1036), hp1117 (jhp1045/hpag1055)*—(prefixes “*hp*” in strain 26695; “*jhp*” in strain J99; “*hpag*” in strain HPAG1) (Figure 1A). Each genome also contains strain-specific *slr* genes: *hp0336* in 26695, and *jhp0318* (similar to *hpag0339*) and *jhp1437* in J99. Reciprocal BLAST analysis revealed sequence similarities with domains of human Sel1L, ranging from 38% with *jhp1437* to 51% with *hp1117* (Figure S1), an intriguing pattern, even though such homologies do not by themselves demonstrate functional equivalence.

A strain of the related H. acinonychis encodes seven SLR homologs [42], six of which are nearly identical in amino acid sequence to SLRs of H. pylori (Figure 1A); this suggests either equivalent selection of *slr* gene–related function in these two species or recent interspecies transfer between H. pylori and *H. acinonychis,* which occurs readily in culture or in vivo [43]. A strain of the related nongastric pathogen H. hepaticus encodes six SLR homologs [44], which are relatively less related to those of *H. pylori*. In contrast, strains from genera most closely related to *Helicobacter, Campylobacter,* and *Wolinella* [45] each contain only one or two *slr* genes (Figure 1A). The *slr* genes are organized into discrete repeating units, which should be prone to duplication events [32]. Amino acid identities of 25%–70% are seen among SLR motifs in different members of this protein family in H. pylori (Table S3). Three of the six *H. hepaticus slr* genes have close homologs in H. pylori (*hh*1827 and *hp0235*; *hh0718* and *hp0628;* and *hh0816* and *hp0519*)*.* These features suggest a possible *Helicobacte*r-lineage–specific *slr* gene family expansion (duplication) after *Helicobacters* diverged from *Campylobacter* and *Wolinella,* and near the time of H. pylori and H. acinonychis versus H. hepaticus divergence. Also tenable is a model of separate gene family expansions in H. pylori and *H. hepaticus,* and even in *H. acinonychis,* since the corresponding *slr* homologs have different chromosomal locations (flanking genes) in these three species (unpublished data).

When a gene family's expansion is adaptive, the sequences of individual members should reflect selective forces that operated during and after this expansion. In general, paralogs that subsequently suffer inactivating mutations tend to be lost from the population over time [32,46]. More important evolutionarily are the paralogs that diverged, and acquired new functions, or optimized or subdivided complex ancestral functions*.* Purifying selection (ω < 1) predominates in the evolution of genes whose roles remained constant, whereas positive selection (ω > 1) predominates in cases of genes whose functions have diverged [32,46]. Accordingly, we determined ω values to test if *slr* gene family expansion was accompanied by functional divergence of paralogs, and, more generally, examined selection pressures that operated on this gene family.

### Positive Selection during *slr* Gene Family Expansion

We applied two codon-based models of sequence evolution to obtain ML estimates of selective pressures during *slr* gene family expansion, starting with sequences of the eight and nine *slr* genes in the three sequenced H. pylori genomes (Figure 1B). The simplest one-ratio model assumes the same ω for all branches, whereas the free-ratio (FR) model allows ω to vary among branches [33]. These models are nested, and hence can be compared using a standard likelihood ratio test (LRT). ML estimates were computed under varying conditions using different sets of initial values for ω and κ to confirm optimal algorithm convergence. Regardless of underlying assumptions, the FR model fit the data significantly better than the M0 model (−*InL*_(FR)_ = 11286.639; −*InL*_(M0)_ = 11356.182; χ^2^ = 140.062, degrees of freedom = 47; *p* < 0.00001; initial ω = 2, κ = 2; equilibrium codon frequencies estimated as free parameters). This suggested that ω varied significantly among individual branches of the *slr* gene-family phylogeny (Figure 1B; Table S4). Strong positive selection was evident in several branches, again indicating that *slr* gene family expansion was driven by selection for functional divergence among paralogs.

Given the multiple *slr* genes in these three *Helicobacter* species, *slr* family expansion might have occurred well before H. pylori became widespread in humans: that is, in ancient non-human hosts, possibly reflecting generalized selection pressures during mucosal colonization. Alternatively, because distributions of *slr* genes in the three sequenced H. pylori genomes vary, these expansions could have been more recent, especially given the ease with which gene duplications can arise [47,48], possibly facilitated in *H. pylori* by its lack of a MutHSL DNA repair system [49], and/or induced by reactive metabolites generated during infection [50]. In either case, evidence of functional divergence among *H. pylori slr* homologs driven by positive selection makes it appealing to imagine their products affecting traits important for H. pylori mucosal colonization. With this perspective, and prompted by the nonrandom geographic distribution of *hp0519* indels, we determined DNA sequences of six *slr* genes present in most East Asian, Western European, and African H. pylori strains using isolates from a representative strain collection.

### Non-Neutral Evolutionary Dynamics of *hp0519*

**Figure 2:**
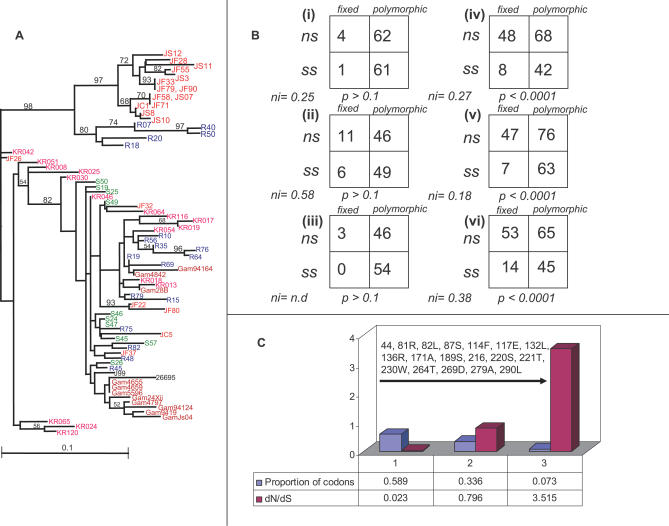
Evolutionary Dynamics of *hp0519* (A) ML phylogenetic tree showing clear separation of Japanese and Korean *hp0519* alleles. The ML tree was reconstructed using an internal 322-bp fragment of *hp0519* (*n* = 76), assuming the TrN + I + Γ substitution model (parameter estimates can be obtained from authors on request). Significant bootstrap support values (>50) are indicated. Origins of H. pylori strains are color-coded: red, Japan; pink, Korea; green, Spain; brown, The Gambia; and blue, South Africa. Bar scale indicates 0.1 nucleotide substitutions per site. (B) Adaptive divergence of Japanese *hp0519* alleles. Pairwise MacDonald-Kreitman tests using polymorphism data from complete *hp0519* DNA sequences (shown in Figure 3A). Pairwise comparisons (i), (ii), and (iii) (Korea versus Europe, Korea versus The Gambia, and The Gambia versus Europe, respectively) revealed a neutral evolutionary dynamic dominated by genetic drift. In contrast, significant deviation (*p* < 0.0001) from neutral evolution was observed in pairwise comparisons (iv), (v), and (vi) (Japan versus Korea, Japan versus Europe, and Japan versus The Gambia, respectively). *ni,* neutrality index, indicates the extent to which the levels of amino acid polymorphism depart from the expected in the neutral model [67]; *ni* = 1 indicates neutral evolution; *ni* > 1 indicates excess amino acid variation within groups, and *ni* < 1 indicates an excess of amino acid evolution between groups. (C) Frequency distribution of three codon classes (p0, p1, and p2) and their associated *d_N_ /d_S_* ratios computed under the SSM M3 for *hp0519*. Codons under positive selection (codon class p2) are shown. Detailed parameter estimates and model comparisons are shown in Table S6B.

Sequences of Japanese and Korean alleles of housekeeping genes are typically intermingled in the same clusters (Figure S2B; and Dailide and Berg, unpublished). Therefore, we asked if *hp0519* single nucleotide polymorphisms (SNPs) also differed geographically by sequencing a 322-bp *hp0519* segment internal to these “24” and “15” indels in 78 strains. These internal *hp0519* sequences contained many SNPs, which fell into separate Japanese (*n =20*) and Korean (*n = 16*) allele clusters (Figure 2A), in accord with the PCR-based indel results. Permutation–randomization tests of this 322-bp internal segment suggested great genetic differentiation, perhaps reflecting separation of Japanese island and Korean mainland populations (F_ST_ = 0.5, p < 0.001; Table S5), and a critical difference in the forces that had operated in these two regions.

**Figure 3:**
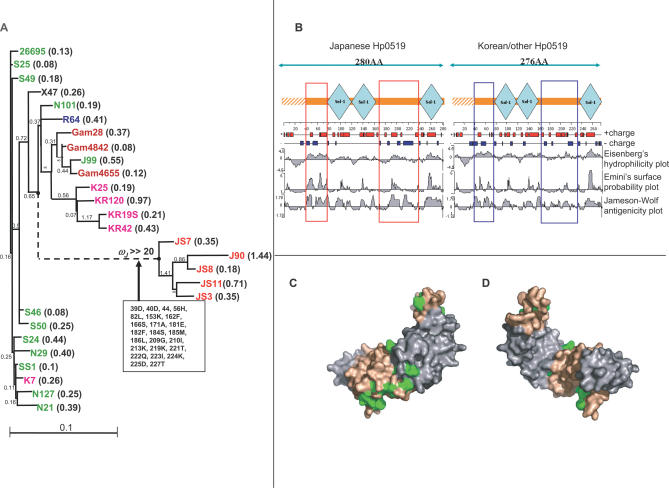
Adaptive Divergence of Japanese and Korean *hp0519* Alleles (A) Identification of Japanese *hp0519* lineage-specific adaptive mutations. Complete nucleotide sequences (873 bp) from 27 representative strains were determined and the input ML tree was computed assuming the TVM + I + Γ (parameters are available from authors on request). Phylogenetic tree shown was computed under the FR model, which fit the data significantly better than the M0 model (*−InL*_(FR_) = 4313.63 and *−InL*_(M0)_ = 4359.72; *χ* ^2^ = 92.12, degrees of freedom = 50, *p* < 0.001). ω values for each lineage are indicated; values for extant taxa are shown in parentheses. Origin of H. pylori strains is color-coded as in Figure 2A; bar scale indicates number of nucleotide substitutions per codon. Codon sites under positive selection in the Japanese lineage (inset) were identified using two LSSMs, M2JM2 and M2JM3; parameter estimates and model comparisons are shown in Table S6B, which is available online. (B) Secondary structure analysis of Japanese-type Hp0519 with Korean-type Hp0519. The rapidly evolving amino acids were localized to boxed regions where they likely affect charge distribution and alter protein hydrophilicity, surface probability, and antigenicity. This analysis was done using PROTEAN package (DNASTAR, WI). (C*−*D) Molecular surface of the Japanese-type Hp0519 homology model. The grey and tan colors refer to regions inside and outside the predicted SLRs, respectively. Green surface areas indicate positively selected residues.

To better understand the evolutionary forces driving this divergence, we next determined full-length *hp0519* sequences (approximately 873 bp) from African, European, and East Asian strains (*n = 27*), chosen randomly from the larger dataset shown in Figure 2A, and reconstructed an ML phylogeny with these data. This revealed Japanese–Korean allele separation (bootstrap support = 100), as expected (Figure 3A). Further pairwise permutation-randomization tests of all 27 full-length *hp0519* sequences also showed genetic differentiation among H. pylori subpopulations in various geographic regions (F_ST_ > 0.5, all comparisons; Table S5). Additional pairwise comparisons of five Korean and five Japanese full-length *hp0519* sequences revealed 56 fixed differences (sites at which all sequences in one population differed from all sequences in a second population), versus 33 polymorphisms shared between them. Both sets showed unique polymorphisms (i.e., sites polymorphic in one set, monomorphic in the other; 68 in Japanese and 42 in Korean, respectively). This inverse relationship between fixed and shared polymorphisms suggests either ancient separation of Korean and Japanese *hp0519* alleles or selection for accelerated accumulation of SNPs in at least one population. Application of the McDonald–Kreitman test for adaptive evolution showed that the ratio of nonsynonymous changes to synonymous changes among fixed differences (48/8) was significantly higher than that among polymorphic differences (68/42) (*p* < 0.001, Fisher's exact test and G-test) (Figure 2B). This outcome suggested that the accumulation of nonsynonymous substitutions in Japanese versus Korean *hp0519* alleles had been driven by positive selection. In contrast, equivalent tests of *hp0519* sequences from other populations suggested divergence between them due mostly to random genetic drift. ML phylogenies of the *hp0518* and *hp0520* genes that flank *hp0519* from six representative Korean and six representative Japanese strains did not show any distinction between these two East Asian populations (Figure S2A). This indicated that divergence of Japanese versus Korean *hp0519* alleles was due to selection on *hp0519* itself, not due to linkage to an even more highly selected gene.

Selection pressures on *hp0519′s* individual codons and branches of its phylogenetic tree were next studied in detail using three groups of codon-based models of sequence evolution and ML-based LRTs: 1) site-specific models (SSMs), which examine variation in selection pressures across codons and assume a single ω across the phylogeny [35]; 2) lineage-specific models (LSMs), which allow ω to vary among lineages, while assuming a single rate across all codons [51]; and 3) lineage-site–specific models (LSSMs), which allow ω to vary both among codons and across the phylogeny [36]. SSMs confidently identified 18 sites under positive selection (ω_2_ = 3.515; Bayesian probability > 0.99) (Figure 2C; Table S6A), which suggested different selective pressures at different sites in *hp0519.* [Equivalent site-specific positive selection was also detected in *hp0519* codons by the single-likelihood ancestor counting (SLAC) and fixed-effects likelihood (FEL) methods hosted at http://www.datamonkey.org (unpublished data)]. Previous work has shown that codon-based models implemented, in M7 and M8 models, in particular, are usually not adversely affected by recombination in bacterial datasets [52–54]. However, because the ML approach used here explicitly assumes a phylogenetic tree when estimating selection pressures, we also assessed if the extensive recombination typically seen in *H. pylori* populations could have produced a false-positive signal for positive selection. This entailed repeating the analysis assuming that sequences were linked by a “star” phylogeny, where lineages diverge simultaneously from a single root node; this removes the effect of phylogenetic history, including recombination events from the outcome. This analysis again indicated positive selection (*p* < 0.0001 for M3 versus M2 and M8 versus M7), with higher ω values under the M3 and M8 models, and with the same sites usually in the positively selected class as in the original analysis (Table S6C).

Next, a two-ratio LSM model, M2J (ω_J_ for Japanese foreground branch; ω_R_ for all other branches (*background lineages*)) was constructed, to test a priori whether ω_J_ was significantly different from ω_R_. M2J fit the data significantly better than M0 (*p* < 0.0001; Table S6B) and suggested that divergence of Japanese *hp0519* lineage was driven by positive selection (ω_J_ = 1.6). In accord with this, the FR model, which assigned an independent ω for each lineage, also fits the data significantly better than M0 (*p* < 0.0001; Table 6B). This suggested that *hp0519* alleles had been subject to significantly different selective pressures in different lineages (Figure 3A). To identify rapidly evolving codons in the Japanese lineage, we constructed two LSSMs, M2JM2 and M2JM3, which assigned a different value to the Japanese lineage (ω_J_) and compared them for fit against M1 and M3 SSMs, respectively. Both LSSMs confidently identified 26 sites that had been strongly selected (ω_J_ >> 20; Table S6B) in the ancestral Japanese lineage. This confirmed that divergence of Korean and Japanese *hp0519* alleles was driven by strong positive selection that had favored specific adaptive changes in Japan. These differences are not explained by models invoking founder effects or random genetic drift alone. We suggest, rather, that this divergence reflects a condition unique to H. pylori in the Japanese islands at some recent evolutionary time. Possibilities include differences between the islands and the East Asian mainland in prevalence of other pathogens or parasites that affect host responses to H. pylori [55,56] and how H. pylori can best manage them; or host genotype, diet or nutrition, or sociocultural features that could also affect host responses to infection. The divergent Japanese-type *hp0519* alleles might have existed at low frequency before being strongly selected in Japan. Alternatively, they might have arisen by more recent stepwise mutation and selection, perhaps only starting when rice-based agriculture was brought to Japan some 2,300 years ago, along with changes in diet, lifestyle, risk of infection, etc.

### Mapping of Adaptive Substitutions on SLR Protein Structure

To examine adaptive evolution in the Japanese *hp0519* lineage in a protein structure–function context, we applied several methods for comparative secondary structure analysis and modeled Hp0519 three-dimensional structure on the experimentally determined crystal structures of its homologs HcpB (Hp0336) [57] and HcpC (Hp1098) [58]. Twenty four of 26 (92%) sites under positive selection were in regions near but not overlapping the predicted SLRs, with 19 of 26 (73%) between the second and third SLR. Two of these adaptive residues (44 and 216) were located within the Δ24 or Δ15 indels, suggesting that these regions were also important functionally, whereas the other adaptive residues were separate from these indels. Strong conservation of Hp0519 SLR motifs suggests that they are critical for protein function. Secondary structure comparisons revealed striking differences between Japanese-type Hp0519 proteins and all others in amino acid charge distribution, hydrophilicity, surface probability, and antigenic index (Figure 3B). Although modelling of Hp0519 structure was complicated by significantly lower sequence conservation and the presence of indels, threading analysis suggested that Hp0519 folds into a three-dimensional structure similar to that of HcpB and HcpC (*z*-score = 31.19; Table S7). Secondary structure analysis localized most sites under positive selection to loop regions, which are generally surface-exposed and potentially positioned to contact cognate host proteins. Data supporting the computational prediction that Hp0519 protein is secreted was obtained by generating plasmids that encode recombinant Hp0519-FLAG fusion proteins with and without predicted signal peptides, and expressing them in BL21 DE3 Escherichia coli cells. Tests using the α-FLAG M2 antibody showed that most Hp0519 encoded by full-length *hp0519* (predicted signal peptide intact) was secreted into the culture supernatant, whereas that encoded by an engineered *hp0519* variant that lacked the signal peptide coding sequence remained with the cell pellet (Figure S3). Expecting equivalent signal peptide–dependent secretion of Hp0519 protein in *H. pylori,* we propose that many of the surface-exposed adaptive changes identified computationally may affect the strength or specificity of Hp0519′s interaction with cognate receptors or other host components. Other surface-exposed Hp0519 residues might have evolved under host immune selection, in particular if certain Hp0519-specific immune responses could inhibit H. pylori growth or persistence. In this last scenario, the directional nature of positive selection, specifically in the Japanese lineage, would suggest unique immunological pressures in Japan at some point in *H. pylori's* evolution. Finally, some of the observed adaptive changes might have been context-dependent, compensating for deleterious mutations elsewhere in the same gene [59–61], which themselves could have accumulated by chance (drift), or been specifically selected at an earlier time, if different selective pressures operated then.

### Most *slr* Genes Evolve Rapidly, Often in Discrete H. pylori Lineages

**Figure 4:**
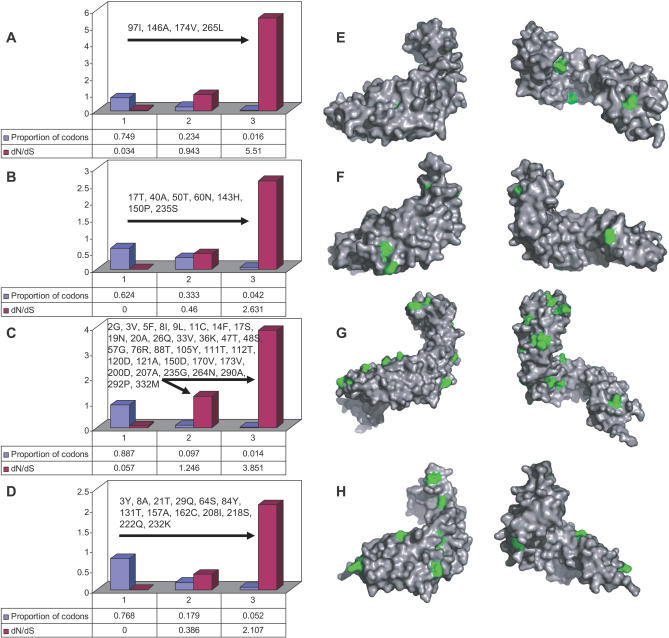
Adaptive Evolution in Other *slr* Genes (A–D) Frequency distribution of codon classes (p0, p1, and p2) and their associated *d_N_*/*d_S_* ratios computed under the SSM M3 for *hp0160*, *hp0211*, *hp0235,* and *hp1117,* respectively. Parameter estimates and model comparisons are shown in Tables S8–S12. (E–H) Mapping of residues under positive selection on Hp0160, Hp0211, Hp0235, and Hp1117 homology models. Convex and concave surfaces are shown on the left and right sides, respectively.

To learn if selection for amino acid sequence change in different populations was common to the *slr* gene family, in general, versus specific to *hp0519,* we sequenced five additional *slr* family members present in nearly all H. pylori strains (*hp0160*, *hp0211*, *hp0235*, *hp0628*, and *hp1117*) from ≥32 isolates variously from East Asia (Japan, Korea), West Europe (Spain, England), and Africa (South Africa, The Gambia). The *slr* gene phylogenies revealed separate clustering of African, European, and East Asian alleles, as is typical of housekeeping genes. In striking contrast to *hp0519,* there was no distinction between Korean and Japanese alleles of these other *slr* genes (Figures S4A–S7A and S8). Analysis of selective pressures by SSMs suggested that each *slr* gene, except *hp0628,* that was analyzed here had experienced different selective pressures at different amino acid sites during their evolution (Figure 4A–4D; Tables S8–S12). Homology-based structural modeling suggested that most sites under positive selection in these *slr* genes also encoded surface-localized amino acids (Figure 4E– 4F). This outcome supports the idea of adaptive sites in SLR proteins potentially affecting their interactions with cognate host components.

LSMs suggested that the different *hp0160, hp0211,* and *hp1117* lineages also evolved at different rates (Figure S4B–S7B). For example, positive selection was more evident in the East Asian and The Gambian *hp0160* lineages (ω = 2.26 and 2.32, respectively), than in South African and European lineages (ω = 0.29 and 0.001, respectively). Similar lineage-specific positive selection was also seen in phylogenies of *hp1117* and of *hp0211;* Hp0211 (HcpA) protein induces a cytokine IL12–mediated pro-inflammatory response [30]. Although SSMs suggested heterogeneous selective pressures and rapid evolution of certain codons in *hp0235*, LSMs indicated that ω did not vary significantly among the lineages studied (Table S11); a sampling of additional populations, however, might identify *hp0235* lineages that had evolved more rapidly. The *hp0628* gene was exceptional, in that SSMs and LSMs indicated evolution dominated by purifying selection (no codon class under positive selection), suggesting that it is functionally constrained, although ω did vary among codon sites (Table S12). Taken together, these outcomes illustrate that H. pylori evolution has not been strictly neutral genome-wide: that amino acid level polymorphisms fixed by positive selection are particularly abundant in certain key genes, perhaps often selected by features of the host milieu that vary geographically. Different sets of selective pressures operated in different members of the *slr* gene family and only on particular sites in any given gene.

### Conclusions

The many decades during which H. pylori can persist in the gastric mucosa despite inflammation and other host defenses, the differences among individuals and geographic regions in the intensity and specificity of host responses, and the changes in responses with age and as infection progresses, all coupled with the possibility of H. pylori exploiting these responses while avoiding clearance by them [23], suggests a need for active response management by the bacterium. It is especially in this framework that we studied the evolutionary dynamics of the *H. pylori slr* gene family. The members of this family encode secreted proteins with homology to the Sel1 group of eukaryotic regulatory proteins that, through their interaction with other eukaryotic proteins, affect cell proliferation, apoptosis, immune response, and intracellular trafficking [62,63]. We found that positive selection played a dominant role in *slr* gene evolution: that different amino acids were selected at particular sites in a given protein in different geographic areas, and that effects were more extreme for some *slr* genes than for others in the populations examined. We suggest that these findings be interpreted in terms of within- and between-host pathogen dynamics: that differences among hosts in physiologic traits had selected for changes in cognate microbial proteins (here H. pylori SLR proteins). Also to be included in this category, we suggest, should be selection for altered interaction with or recognition by critical components of the host immune system, which can differ among humans genetically or physiologically, reflecting factors such as infectious disease history, nutrition, stress, etc. Successful adaptation to these forces should also contribute to the extraordinary chronicity of H. pylori infection. Geographic differences in predominant H. pylori–associated diseases noted above [13,14,64] are likely due to multiple factors that may include infections by other pathogens that affect responses to H. pylori infection, diet, and human genotype [3,15]. It is tempting to consider these trends also being affected by several aspects of H. pylori genotype, including predominant allele types of *slr* genes.

#### A model for H. pylori evolution.

Emerging data suggest that genetic drift and positive selection each affect the evolutionary history of H. pylori populations, with genetic drift being a major force at most loci, but with selection for change in amino acid sequence being relatively important at certain key loci and at certain times. Genetic drift should allow H. pylori subpopulations to explore various possible gastric physiologic niches (formally, adaptive landscapes), e.g., as may be imposed by differences in human physiology; and natural selection helps fix particular mutant or recombinant genotypes that may be better suited to features of the local host population [16]. Beneficial alleles tend to be maintained by selection until the environment (adaptive landscape) changes such that they no longer contribute to fitness. An important prediction of this thinking is that H. pylori genes that show signs of positive selection (i.e., ω > 1) in specific lineages may encode products for which changes in the specificity or strength of interaction have been selected in particular locales and times in evolution. Just what host functions are targeted by SLR proteins, especially by Hp0519, now merits detailed analysis. Especially with Hp0519, we are seeking to identify interacting host proteins, and to examine the impact of its variant forms on these interactions and on colonization or virulence. More generally, further search for, and analysis of, *H. pylori's* determinants that have been subject to positive selection in particular populations should provide new understanding of mechanisms important in establishment and maintenance of chronic infection and disease, and perhaps in effective management or eradication of these infections in human populations worldwide.

## Materials and Methods

### Bacterial isolates and molecular methods.

H. pylori isolates were obtained from phylogenetically distinct West European (Spain, England), African (The Gambia, South Africa), and East Asian (Japan, Korea) populations and have been described earlier [8,52]. All isolates were obtained from patients with gastric complaints who had undergone diagnostic endoscopy with informed consent. Standard methods were used for H. pylori propagation and storage. Primers used for PCR and sequencing are listed in Table S13. Standard methods were used for genomic DNA preparations, PCRs, and sequencing.

### Computations.

Nucleotide diversities within and between population, F*_ST_* and permutation tests, and McDonald–Kreitman tests were done with DNASP version 4.1 (http://www.ub.es./dnasp). Phylogenetic reconstruction using *slr* gene sequences was performed with the ML approach implemented in PAUP*4b10 (http://paup.csit.fsu.edu/). An ML phylogeny was reconstructed under the best-fit model [determined with MODELTEST, version 3.7 (http://darwin.uvigo.es/software/modeltest.html)] by using a combination of heuristic searches and branch swapping to further optimize the likelihood score and substitution parameters. The significance of observed phylogenetic groupings was assessed by a bootstrap analysis performed with 1,000 replicates under the distance optimality criterion, while incorporating ML-optimized model and parameters. Phylogenetic trees were visualized with TreeView version 1.6.6 (http://taxonomy.zoology.gla.ac.uk/rod/treeview.html). The selective pressures operating on *H. pylori slr* genes were measured using an ML method that takes into account the sequence phylogeny and assesses the fit to the data of various models of codon evolution that differ in how ω varies across the sequence or across the phylogeny [33]. Three classes of codon-based analysis were used in this study: (a) SSM analysis, whereby models (M0, M1, M2, M3, M7, and M8) assume a single ω for all branches of the tree, but allow ω to vary among individual codon sites, thereby providing a measure of heterogeneity in selection pressures acting across the gene sequence [35]; M7 and M8 perform robustly even when recombination has occurred; (b) LSM analysis, wherein models (FR, two-ratio, etc.) assume that ω varies among individual branches of the phylogeny, but that all codon sites are under the same selective pressure, thereby providing a measure of selective pressures acting on the gene in different lineages [51]; and (c) LSSM analysis, which allows ω to vary simultaneously among sites and lineages [36]. Positive selection was inferred when codons with ω of >1 were identified and the likelihood score (*−InL*) of the codon substitution model in question was significantly higher than the likelihood of a nested model that did not take positive selection into account. The probability that a specific codon belonged to the neutral, negative, or positively selected class was calculated by using Bayesian methods implemented in PAML version 3.14 (http://abacus.gene.ucl.ac.uk/software/paml.html). Multiple runs, assuming different initial ω and κ values, and different models for estimating equilibrium codon frequencies (calculated from the average nucleotide frequencies at the three codon positions tables (F3X4) or used as free parameters) were analyzed for each gene to verify the convergence optima for each model.

Homology modeling of Hp0160, Hp0211, Hp0235, Hp0519, and Hp1117 started with a multiple sequence alignment including the protein sequences of the template structures HcpB (1KLX) [57] and HcpC (1OUV) [58] using program CLUSTALW. Due to the modular architecture of Hcps, different superpositions of HcpB and HcpC are possible and meaningful. This molecular feature increased conformational space and allowed modeling of protein sequences that were significantly longer than the template structures. The resulting structure-based sequence alignment was merged with the multiple sequence alignment. The alignment was manually curated taking into account the predicted secondary structure [program JPRED (http://www.compbio.dundee.ac.uk/∼www-jpred)]. Homology models were generated using program MODELLER (http://salilab.org/modeller/modeller.html). To identify distant structural homologues of Hp0519, its protein sequence was threaded against databases of protein structures using the programs FUGUE (http://www-cryst.bioc.cam.ac.uk/fugue) and LOOPP (http://cbsu.tc.cornell.edu/software/loopp/). Figures were generated with PYMOL (http://www.pymol.org).

## Supporting Information

Figure S1 Sequence Similarity of *H. pylori slr* Genes (Query) with Human Sel1L (sbjct) Reciprocal BLAST analysis was done using SMART (http://smart.embl-heidelberg.de/), and figures were generated with BLAST2P (http://www.ncbi.nlm.nih.gov/BLAST).
(355 KB PDF)

Figure S2 ML Phylogenies of *hp0518* and *hp0520* (A) and The Neutral Population Structure of H. pylori Strains Included in This Study (B) (A) Full-length sequences for *hp0518* (393 bp) and *hp0520* (*993 bp*) were determined from 16 representative East Asian and Spanish isolates using primers listed in Table S13. Phylogenies were reconstructed assuming the GTR +I + Γ substitution model and were further optimized using ML for among and within-site rate variation (optimized parameters for both phylogenies are available from the authors upon request). Significant bootstrap values (≥50) are shown. Phylogenies are unrooted. Bar scale = 0.01 nucleotide substitutions per site.
(B) Nucleotide sequence from six housekeeping genes, (*atpA, cysS, recA, ppa, glr,* and *glmM*) were concatenated to yield a sequence length of 3,885 bp. A neighbor-joining tree was reconstructed using Kimura-2 parameter distance as implemented in MEGA version 3.1 (http://www.megasoftware.net). Shaded area includes Japanese and Korean strains, which were clearly intermingled and formed a single cluster.
(1.3 MB EPS)

Figure S3 Secretion of Recombinant Hp0519-FLAG Fusion Protein in E. coli BL21 DE3 (A) Predicted domain architecture of Hp0519: filled area (blue) indicates predicted signal peptide (SP); hatched areas show SLRs. Two pBS II KS+ plasmid-based constructs of recombinant Hp0519-FLAG fusion proteins were generated using standard PCR-based protocols: construct C1, with intact signal peptide and containing a C-terminus FLAG fusion; construct C2, predicted mature Hp0519 coding region and C-terminal FLAG fusion. The FLAG tag is an eight amino acid peptide (DYKDDDDK) that, because of its small size, generally does not interfere with protein folding or activity.
(B) IPTG induction of C1 and C2 expression in BL21 DE3 cell pellets; UI-uninduced controls; expression was confirmed by standard Western blotting with α-FLAG M2 monoclonal antibody.
(C) Expression of C1 and C2 constructs in growth culture supernatants—only C1 product cloned with signal peptide was detected in cell supernatants and was confirmed by Western blotting.
(375 KB PDF)

Figure S4 ML Phylogeny of *hp1117* (*N = 33*) (A) and ML Estimation of Selection Pressures Acting on Each Individual *hp1117* Lineage (B) (A) The phylogeny was reconstructed assuming the GTR + Γ substitution model and was optimized to the following parameters using heuristic searches and a tree bisection–reconnection algorithm: rate matrix: A → C = 1.56, A → G = 7.18, A → T = 0.054, C → G = 0.57, C → T = 9.75, and G → T = 1; base frequencies: A = 0.33, C = 0.12, G = 0.26; and Γ distribution shape parameter, α = 0.178. Significant bootstrap values (≥50) are shown. Phylogeny is unrooted. Bar scale = 0.01 nucleotide substitutions per site.
(B) Phylogenetic tree shown was computed with the FR model estimating equilibrium codon frequencies as free parameters. Parameter estimates are shown in Table S8. Arrows indicate branches that experienced positive selection, and corresponding ω values are shown. ω = ∞ indicates branches that accumulated mostly nonsynonymous substitutions during their divergence; ω = # indicates branches that experienced strong purifying selection (ω < 0.0001); and ω for extant taxa is shown in parentheses. Phylogeny is rooted using the out-group method, implemented in PAUP4b10, for the purpose of clarity. Origin of H. pylori strains is color-coded: green, Korea; pink, Japan; blue, Spain; brown, The Gambia; and orange, South Africa. Bar scale indicates nucleotide substitutions per codon.
(122 KB PDF)

Figure S5 ML Phylogeny of *hp0235* (N = 32) (A) and ML Estimation of Selection Pressures Acting on Each Individual *hp0235* Lineage (B) (A) The phylogeny was reconstructed assuming the GTR + I + Γ substitution model and was optimized to the following parameters using heuristic searches and a tree bisection–reconnection algorithm: rate matrix: A → C = 2.03, A → G = 7.02, A → T = 0.52, C → G = 1.69, C → T = 15.9, and G → T = 1; base frequencies: A = 0.31, C = 0.15, G = 0.25, proportion of invariable sites, I = 0.29; and Γ distribution shape parameter, α = 0.33. Significant bootstrap values (≥50) are shown. Phylogeny is unrooted. Bar scale = 0.01 nucleotide substitutions per site.
(B) The phylogenetic tree shown was computed with the FR model estimating equilibrium codon frequencies as free parameters. Parameter estimates are shown in Table S9. Although SSMs had suggested heterogeneous selective pressures among *hp0235* codons, the FR model did not show a better fit than the one-ratio M0 model (Table S9), indicating that ω did not vary significantly among different branches. Phylogeny is rooted using the out-group method, implemented in PAUP4b10, for the purpose of clarity. See footnotes to Figure S4 for more details. Bar scale indicates nucleotide substitutions per codon.
(257 KB PDF)

Figure S6 ML Phylogeny of *hp0211* (*N=34*) (A) and ML Estimation of Selection Pressures Acting on Each Individual *hp0211* Lineage (B) (A) The phylogeny was reconstructed assuming the GTR + I + Γ substitution model and was optimized to the following parameters using heuristic searches and a tree bisection–reconnection algorithm: rate matrix: A → C = 1.98, A → G = 5.51, A → T = 0.37, C → G = 0.081, C → T = 11.9, and G → T = 1; base frequencies: A = 0.33, C = 0.13, G = 0.26, proportion of invariable sites, I = 0.28; and Γ distribution shape parameter, α = 0.36. Significant bootstrap values (≥50) are shown. Phylogeny is unrooted. Bar scale = 0.01 nucleotide substitutions per site.
(B) Phylogenetic tree shown was computed with the FR model estimating equilibrium codon frequencies as free parameters. Parameter estimates are shown in Table S10. Arrows indicate branches that experienced positive selection, and corresponding ω values are shown. ω = ∞ indicates branches that accumulated mostly nonsynonymous substitutions during their divergence; ω = # indicates branches that experienced strong purifying selection (ω < 0.0001); and ω for extant taxa is shown in parentheses. Phylogeny is rooted using the out-group method, implemented in PAUP4b10, for the purpose of clarity. Origin of H. pylori strains is color-coded: green, Korea; pink, Japan; blue, Spain; brown, The Gambia; and orange, South Africa. Bar scale indicates nucleotide substitutions per codon.
(213 KB PDF)

Figure S7 ML Phylogeny of *hp0160* (*N=33*) (A) and ML Estimation of Selection Pressures Acting on Each Individual *hp160* Lineage (B) (A) The phylogeny was reconstructed assuming the GTR + I + Γ substitution model and was optimized to the following parameters using heuristic searches and a tree bisection–reconnection algorithm: rate matrix: A → C =1.47, A → G = 6.76, A → T = 0.23, C → G = 0.731, C → T = 16.5, and G → T = 1; base frequencies: A = 0.30, C = 0.12, G = 0.26, proportion of invariable sites, I = 0.38; and Γ distribution shape parameter, α = 0.46. Significant bootstrap values ( ≥50) are shown. Phylogeny is unrooted. Bar scale = 0.01 nucleotide substitutions per site.
(B) Phylogenetic tree shown was computed with the FR model estimating equilibrium codon frequencies as free parameters. Parameter estimates are shown in Table S11. Arrows indicate branches that experienced positive selection, and corresponding ω values are shown. ω = ∞ indicates branches that accumulated mostly nonsynonymous substitutions during their divergence; ω = # indicates branches that experienced strong purifying selection (ω < 0.0001); and ω for extant taxa is shown in parentheses. Phylogeny is rooted using the out-group method, implemented in PAUP4b10, for the purpose of clarity. Origin of H. pylori strains is color-coded: green, Korea; pink, Japan; blue, Spain; brown, The Gambia; and orange, South Africa. Bar scale indicates nucleotide substitutions per codon.
(395 KB PDF)

Figure S8 ML Phylogeny of *hp0628* (*n =* 34) The phylogeny was reconstructed assuming the GTR + I + Γ substitution model and was optimized to the following parameters using heuristic searches and a tree bisection–reconnection algorithm: rate matrix: A → C = 1.97, A → G = 9.12, A → T = 0.60, C → G = 1.13, C → T = 17.9, and G → T = 1; base frequencies: A = 0.28, C = 0.17, G = 0.26, proportion of invariable sites, I = 0.457; and Γ distribution shape parameter, α=0.386. Significant bootstrap values ( ≥50) are shown. Phylogeny is unrooted. Bar scale = 0.01 nucleotide substitutions per site. ML analysis of selection pressures revealed that *hp0628* was under strong functional constraint (purifying selection) (Table S12).
(116 KB PDF)

Table S1 Signal Peptide Prediction for H. pylori SLR Proteins (39 KB DOC)

Table S2 Confidently Predicted Domains, Repeats, Motifs, and Features Using SMART (273 KB DOC)

Table S3 Pairwise Amino Acid Sequence Identity among H. pylori Strain HPAG1 SLR Repeats (32 KB DOC)

Table S4 d_N_ and d_S_ for Each Branch of *slr* Gene-Family Phylogeny Computed with the FR Model (107 KB DOC)

Table S5 Population Differentiation at *hp0519* Locus (34 KB DOC)

Table S6 ML Parameter Estimates (A) ML parameter estimates of selection pressures acting on Hp0519 codons.
(B) ML parameter estimates for episodic adaptive evolution in the Japanese HP0519 lineage.
(C) ML parameter estimates of selection pressures acting on Hp0519 codons assuming a star phylogeny.
(397 KB DOC)

Table S7 Results for Hp0519 Homology Models for European and Japanese Lineages (35 KB DOC)

Table S8 ML Parameter Estimates of Selection Pressures Acting on H. pylori Hp1117 SLR Protein (39 KB DOC)

Table S9 ML Parameter Estimates of Selection Pressures Acting on H. pylori Hp0211 SLR Protein (39 KB DOC)

Table S10 ML Parameter Estimates of Selection Pressures Acting on H. pylori Hp0160 SLR protein (39 KB DOC)

Table S11 ML Parameter Estimates of Selection Pressures Acting on H. pylori Hp0235 SLR Protein (40 KB DOC)

Table S12 ML Parameter Estimates of Selection Pressures Acting on H. pylori Hp0628 SLR Protein (39 KB DOC)

Table S13 Primers Used in This Study (55 KB DOC)

### Accession Numbers

GenBank (http://www.ncbi.nlm.nih.gov/Genbank) accession numbers for sequences generated in this study are from EF372636 to EF372923.

## Abbreviations

LSSM: lineage-site–specific model
ML: maximum likelihood
SLR: Sel1-like repeat
SMART: simple modular architecture research tool
SSM: site-specific model
