# Supplementary material for: Helicobacter pylori Evolution: Lineage- Specific Adaptations in Homologs of Eukaryotic Sel1-Like Genes
Source: PLoS Comput Biol. 2007 Aug 10;3(8):e151. doi: 10.1371/journal.pcbi.0030151 (PMC1941758; doi:10.1371/journal.pcbi.0030151)
Supplement: Supplementary file 1 [file pcbi.0030151.sg001.pdf]

a. Query: Hp0519

```
Score = 46.6 bits (109),  Expect = 0.002
 Identities = 53/237 (22%), Positives = 96/237 (40%), Gaps = 36/237 (15%)

Query  62   QGQYQQAFKDFRLAQEYSIGLGSVYLAKMYLEGKGVKVDYKKAQFYAENAIKGYGSGLLG  121
            Q  Y  A K F+ A E     G + L  MY  G GVK DYK+A  Y   A +G   G +
Sbjct  464  QVNYDLALKYFQKAAEQGWVDGQLQLGSMYYNGIGVKRDYKQALKYFNLASQG---GHIL  520

Query  122  GALILGRMQAEGLGMKKDLKQALKTYRHVVR--MFSNKSTNFANNFRLP--NLAEFTSML  177
                L +M A G G+ +    A++ +++V     +S +     N+++    N A    +L
Sbjct  521  AFYNLAQMHASGTGVMRSCHTAVELFKNVCERGRWSERLMTAYNSYKDGDYNAAVIQYLL  580

Query  178  IGSRFIDLSGLSANPIKFGKKFGILVKKSTQIKDKTLLWEDIAEISSNITLLK-------  230
            +  +  +++  +A  I   ++  I+ +  T  +   L W   A     +  +K
Sbjct  581  LAEQGYEVAQSNAAFILDQREASIVGENETYPR-ALLHWNRAASQGYTVARIKLGDYHFY  639

Query  231  ---------------------QQMGEILYRIGIAYKEGLGTRKKKDRAKKFLQKSAE  266
                                 Q   + ++ +G  +++GLG ++    AK+F   +AE
Sbjct  640  GFGTDVDYETAFIHYRLASEQQHSAQAMFNLGYMHEKGLGIKQDIHLAKRFYDMAAE  696
```

b. Query: Hp0160

```
Score = 70.5 bits (171),  Expect = 1e-10
 Identities = 61/224 (27%), Positives = 92/224 (41%), Gaps = 41/224 (18%)

Query  52   KRSCNLRVGVGCTSLGSMYEDGDGVDQNITKAVFYYRRGCNLRNHLACASLGSMYEDG-D  110
            K     +VG+G   L      G GV+QN  +A  Y+    N  N  A A LG MY +G D
Sbjct  370  KGDVQAQVGLGQLHL----HGGRGVEQNHQRAFDYFNLAANAGNSHAMAFLGKMYSEGSD  425

Query  111  GVQKNLPKAIYYYRRGCHLKGGVSCGSLGFMYFNGTGVKQNYAKALFLSKYACSLNYGIS  170
             V ++   A++Y+++   +   V    LG  Y  G GV+ NY  AL   + A    +
Sbjct  426  IVPQSNETALHYFKKAADMGNPVGQSGLGMAYLYGRGVQVNYDLALKYFQKAAEQGWVDG  485

Query  171  CNFVGYMYRNAKGVQKDLKKALANFKRGCHLKDGASCVSLGYMYEVGMDVKQNGEQALNL  230
               +G MY N  GV++D K+AL  F                                 NL
Sbjct  486  QLQLGSMYYNGIGVKRDYKQALKYF---------------------------------NL  512

Query  231  YKKGCYLKRGSGCHNVAVMYYTGKGVPKDLDKAISYYKKGCTLG  274
              +G ++      +N+A M+ +G GV +    A+  +K  C  G
Sbjct  513  ASQGGHIL---AFYNLAQMHASGTGVMRSCHTAVELFKNVCERG  553
```

c. Query: Hp0211

```
Score = 65.9 bits (159),  Expect = 2e-09
 Identities = 48/190 (25%), Positives = 84/190 (44%), Gaps = 21/190 (11%)

Query  42   KQDFAQAKTHFEKACELKNGFGCVFLGAFYEEGKG-VGKDLKKAIQFYTKGCELNDGYGC  100
            +Q+  +A  +F  A    N     FLG  Y EG   V +  + A+ ++ K  ++ +  G
Sbjct  391  EQNHQRAFDYFNLAANAGNSHAMAFLGKMYSEGSDIVPQSNETALHYFKKAADMGNPVGQ  450

Query  101  NLLGNLYYNGQGVSKDAKKASQYYSKACDLNHAEGCMVLGSLHHYGVGTPKDLRKALDLY  160
            + LG  Y  G+GV  +   A +Y+ KA +    +G + LGS+++ G+G  +D ++AL  +
Sbjct  451  SGLGMAYLYGRGVQVNYDLALKYFQKAAEQGWVDGQLQLGSMYYNGIGVKRDYKQALKYF  510

Query  161  EKACDLKDSPGCINAGYIYS----VTKNFKEAIVRYSKACE--------------LKDGR  202
              A          N   +++    V ++   A+  +   CE               KDG
Sbjct  511  NLASQGGHILAFYNLAQMHASGTGVMRSCHTAVELFKNVCERGRWSERLMTAYNSYKDGD  570

Query  203  GCYNLGVMQY  212
              YN  V+QY
Sbjct  571  --YNAAVIQY  578
```

d. Query: Hp0336

```
Score = 49.3 bits (116),  Expect = 5e-05
 Identities = 41/148 (27%), Positives = 65/148 (43%), Gaps = 16/148 (10%)

Query  3    GGGTVKKDLKKAIQYYVKACELNEMFGCLSLVS------NSQINKQKLFQYLSKACELNS  56
            G   V +  + A+ Y+ KA ++    G   L          Q+N     +Y  KA E
Sbjct  423  GSDIVPQSNETALHYFKKAADMGNPVGQSGLGMAYLYGRGVQVNYDLALKYFQKAAEQGW  482

Query  57   GNGCRFLGDFYENGKYVKKDLRKAAQYYSKACGLNDQDGCLILGYK----QYAGKGVVKN  112
             +G   LG  Y NG  VK+D ++A +Y++ A     Q G ++  Y       +G GV+++
Sbjct  483  VDGQLQLGSMYYNGIGVKRDYKQALKYFNLA----SQGGHILAFYNLAQMHASGTGVMRS  538

Query  113  EKQAVKTFEKACRLG--SEDACGILNNY  138
               AV+ F+  C  G  SE      N+Y
Sbjct  539  CHTAVELFKNVCERGRWSERLMTAYNSY  566
```

e. Query: Hp0235

```
 Score = 96.7 bits (239),  Expect = 2e-18
 Identities = 80/322 (24%), Positives = 134/322 (41%), Gaps = 20/322 (6%)

Query  38   YKSGNYSKATSYFKKACNDGVSEGCTQLGIIYENGQGTRIDYKKALEYYKTACQADDREG  97
            Y   N   A   F+K   +G  +G T LG +Y +G G      KAL YY        +
Sbjct  234  YLPQNIQAAREMFEKLTEEGSPKGQTALGFLYASGLGVNSSQAKALVYYTFGALGGNLIA  293

Query  98   CFGLGGLYDEGLGTTQNYQEAIDAYAKACVLKHPESCYNL--GIIYDR-----KIKGNA-  149
               LG  Y  G+G  Q+ + A+  Y    V  H  S  +L  G +  R     +++
Sbjct  294  HMVLGYRYWAGIGVLQSCESALTHYR--LVANHVASDISLTGGSVVQRIRLPDEVENPGM  351

Query  150  ------DQAVTYYQKSCNFDMAKGCYVLGVAYEKGFLEVKQSNHKAVIYYLKACRLDDGQ  203
                  +  + YYQ        +    LG  +  G   V+Q++ +A  Y+  A    +
Sbjct  352  NSGMLEEDLIQYYQFLAEKGDVQAQVGLGQLHLHGGRGVEQNHQRAFDYFNLAANAGNSH  411

Query  204  ACRALGSLFENGDAGLDEDFEVAFDYLQKACGLNNSGGCASLGSMYMLGRYVKKDPQKAF  263
            A   LG ++  G   + +  E A  Y +KA  + N  G + LG  Y+ GR V+ +   A
Sbjct  412  AMAFLGKMYSEGSDIVPQSNETALHYFKKAADMGNPVGQSGLGMAYLYGRGVQVNYDLAL  471

Query  264  NFFKQACDMGSAVSCSRMGFMYSQGDAVPKDLRKALDNYERGCDMGDEVGCFALAGMYYN  323
             +F++A + G      ++G MY  G  V +D ++AL  +      G  +  + LA M+ +
Sbjct  472  KYFQKAAEQGWVDGQLQLGSMYYNGIGVKRDYKQALKYFNLASQGGHILAFYNLAQMHAS  531

Query  324  ----MKDKENAIMIYDKGCKLG  341
                M+    A+ ++   C+ G
Sbjct  532  GTGVMRSCHTAVELFKNVCERG  553
```

f. Query: Hp0628

```
 Score = 81.6 bits (200),  Expect = 7e-14
 Identities = 76/284 (26%), Positives = 124/284 (43%), Gaps = 21/284 (7%)

Query  81   GDY-----QGAFKLFSQSCDNGNAAGCFAVGAMYANGVGIQTNRLKAARYYEMGCSGGDA  135
            GDY     Q A ++F +  + G+  G  A+G +YA+G+G+ +++ KA  YY  G  GG+
Sbjct  232  GDYLPQNIQAAREMFEKLTEEGSPKGQTALGFLYASGLGVNSSQAKALVYYTFGALGGNL  291

Query  136  TACANLAQMYENKKNADSNDKENALQLYA---------VACQGGDMLACNNLGWMFAN--  184
             A   L   Y           E+AL  Y          ++  GG ++    L     N
Sbjct  292  IAHMVLGYRYWAGIGV-LQSCESALTHYRLVANHVASDISLTGGSVVQRIRLPDEVENPG  350

Query  185  -GSGVPKDYYKAMSYYKFSCENGNDMGCYNLGLMSNVNNIYGIDKAQLSQVDLNYLACNA  243
              SG+ ++    + YY+F  E G+      LG + +++   G+++      D   LA NA
Sbjct  351  MNSGMLEE--DLIQYYQFLAEKGDVQAQVGLGQL-HLHGGRGVEQNHQRAFDYFNLAANA  407

Query  244  GDMMGCANLGWIYANGDLGAPLNNHYAAKYFQMACDGGILGSCNNLGVLYQKGLGVPQDD  303
            G+    A LG +Y+ G    P +N  A  YF+ A D G     + LG+ Y  G GV  +
Sbjct  408  GNSHAMAFLGKMYSEGSDIVPQSNETALHYFKKAADMGNPVGQSGLGMAYLYGRGVQVNY  467

Query  304  QRALDLFSYACDNGFESSCRNYGNFKEHLLHVNPNYGRLFMPYN  347
              AL  F  A + G+       G+   + + V  +Y +    +N
Sbjct  468  DLALKYFQKAAEQGWVDGQLQLGSMYYNGIGVKRDYKQALKYFN  511
```

g. Query: Hp1098

```
Score = 87.8 bits (216),  Expect = 7e-16
 Identities = 67/214 (31%), Positives = 108/214 (50%), Gaps = 13/214 (6%)

Query  57    DLKENSGCFNLGVLYYQGQGVEKNLKKAASFYAKACDLNYSNGCHLLGNLY-YSGQGVSQ  115
             D  EN G  N G+L       E++L +   F A+  D+    G   LG L+ + G+GV Q
Sbjct  344   DEVENPG-MNSGML-------EEDLIQYYQFLAEKGDVQAQVG---LGQLHLHGGRGVEQ  392

Query  116   NTNKALQYYSKACDLKYAEGCASLGGIYHDGK-VVTRDFKKAVEYFTKACDLNDGDGCTI  174
             N  +A  Y++ A +   +   A LG +Y +G  +V +  + A+ YF KA D+ +  G +
Sbjct  393   NHQRAFDYFNLAANAGNSHAMAFLGKMYSEGSDIVPQSNETALHYFKKAADMGNPVGQSG  452

Query  175   LGSLYDAGRGTPKDLKKALASYDKACDLKDSPGCFNAGNMYHHGEGATKNFKEALARYSK  234
             LG  Y  GRG   +   AL  + KA +     G    G+MY++G G  +++K+AL  ++
Sbjct  453   LGMAYLYGRGVQVNYDLALKYFQKAAEQGWVDGQLQLGSMYYNGIGVKRDYKQALKYFNL  512

Query  235   ACELENGGGCFNLGAMQYNGEGVTRNEKQAIENF  268
             A +  +    +NL  M  +G GV R+   A+E F
Sbjct  513   ASQGGHILAFYNLAQMHASGTGVMRSCHTAVELF  546
```

h. Query: Hp1117

```
Score = 86.3 bits (212),  Expect = 2e-15
 Identities = 59/211 (27%), Positives = 100/211 (47%), Gaps = 15/211 (7%)

Query  43    NKEYERAASFYKSAIKNGEPLAYVLLGIMYENGRGVPKDEKKAAEYFQKAVDNDIPRGYN  102
             N    +A  +Y      G  +A+++LG  Y  G GV +  + A  ++ + V N +    +
Sbjct  272   NSSQAKALVYYTFGALGGNLIAHMVLGYRYWAGIGVLQSCESALTHY-RLVANHVASDIS  330

Query  103   NLGVMYKEGRGVPKD-----------EKKAVEYFRIATEKGYTNAYINLGIMYMEG-RGV  150
               G    +   +P +           E+  ++Y++   EKG   A + LG +++ G RGV
Sbjct  331   LTGGSVVQRIRLPDEVENPGMNSGMLEEDLIQYYQFLAEKGDVQAQVGLGQLHLHGGRGV  390

Query  151   PSNYVKATECFRKAMHKGNVEAYILLGDIYYSGNDQLGIEPDKDKAIVYYKMAADMSSSR  210
               N+ +A + F  A + GN  A   LG +Y  G+D   +    + A+ Y+K AADM +
Sbjct  391   EQNHQRAFDYFNLAANAGNSHAMAFLGKMYSEGSDI--VPQSNETALHYFKKAADMGNPV  448

Query  211   AYEGLAESYQYGLGVEKDKKKAEEYMQKACD  241
                GL  +Y YG GV+ +   A +Y QKA +
Sbjct  449   GQSGLGMAYLYGRGVQVNYDLALKYFQKAAE  479
```

i. Query: jhp0318

```
Score = 69.7 bits (169),  Expect = 2e-10
 Identities = 55/200 (27%), Positives = 92/200 (46%), Gaps = 4/200 (2%)

Query  43    VEKNLIKAAQPYTKACELKEGLGCERLWSLYYYGRGVEKNLIKAAQYASKACDLNNGVGC  102
             +E++LI+  Q   +  +++  +G  +L    + GRGVE+N  +A  Y + A +  N
Sbjct  356   LEEDLIQYYQFLAEKGDVQAQVGLGQLH--LHGGRGVEQNHQRAFDYFNLAANAGNSHAM  413

Query  103   KNLGFLYEYGEGVEKDLIKAA-QYASKACDLNNGSGCDVLGFLYGSGKGVEKNLTKAAYF  161
               LG +Y  G  +     + A  Y  KA D+ N  G   LG  Y  G+GV+ N   A  +
Sbjct  414   AFLGKMYSEGSDIVPQSNETALHYFKKAADMGNPVGQSGLGMAYLYGRGVQVNYDLALKY  473

Query  162   YSKACDLNEGLGCFNLGGLY-NGQGVEKDLTKVAYLYSKACELKESFGCGALAVLYINGQ  220
             + KA +     G   LG +Y NG GV++D  +    ++ A +         LA ++ +G
Sbjct  474   FQKAAEQGWVDGQLQLGSMYYNGIGVKRDYKQALKYFNLASQGGHILAFYNLAQMHASGT  533

Query  221   GVEKDLTKADQYISKACKLG  240
             GV +    A +     C+ G
Sbjct  534   GVMRSCHTAVELFKNVCERG  553
```

j. Query: Jhp1437

```
Score = 46.6 bits (109),  Expect = 0.001
 Identities = 47/204 (23%), Positives = 78/204 (38%), Gaps = 44/204 (21%)

Query  43   DKNFTRAKKYFEKACKSNDADGCAILREVYSSGKAIARENARESIEKALEHTATAKVCKL  102
            ++N  RA  YF  A  + ++   A L ++YS G  I  ++
Sbjct  391  EQNHQRAFDYFNLAANAGNSHAMAFLGKMYSEGSDIVPQSN-------------------  431

Query  103  NDAEKCKDLAEFYFNVNDLKNALEYYSKSCKLNNVEGC--MLSATFYNDMIKGLKKDKK-  159
                                + AL Y+ K+  + N  G   +  A  Y    +G++ +
Sbjct  432  -------------------ETALHYFKKAADMGNPVGQSGLGMAYLYG---RGVQVNYDL  469

Query  160  DLEYYSKACELNNGGGCSKLGGDYFFGEGVTKDFKKAFEYSAKACELNDAKGCYALAAFY  219
             L+Y+ KA E     G  +LG  Y+ G GV +D+K+A +Y   A +       Y LA  +
Sbjct  470  ALKYFQKAAEQGWVDGQLQLGSMYYNGIGVKRDYKQALKYFNLASQGGHILAFYNLAQMH  529

Query  220  NEGKGVAKDEKQTTENLEKSCKLG  243
              G GV +      E  +  C+ G
Sbjct  530  ASGTGVMRSCHTAVELFKNVCERG  553
```
