# Supplementary material for: Helicobacter pylori Evolution: Lineage- Specific Adaptations in Homologs of Eukaryotic Sel1-Like Genes
Source: PLoS Comput Biol. 2007 Aug 10;3(8):e151. doi: 10.1371/journal.pcbi.0030151 (PMC1941758; doi:10.1371/journal.pcbi.0030151)
Supplement: Supplementary file 2 [file pcbi.0030151.sg003.pdf]

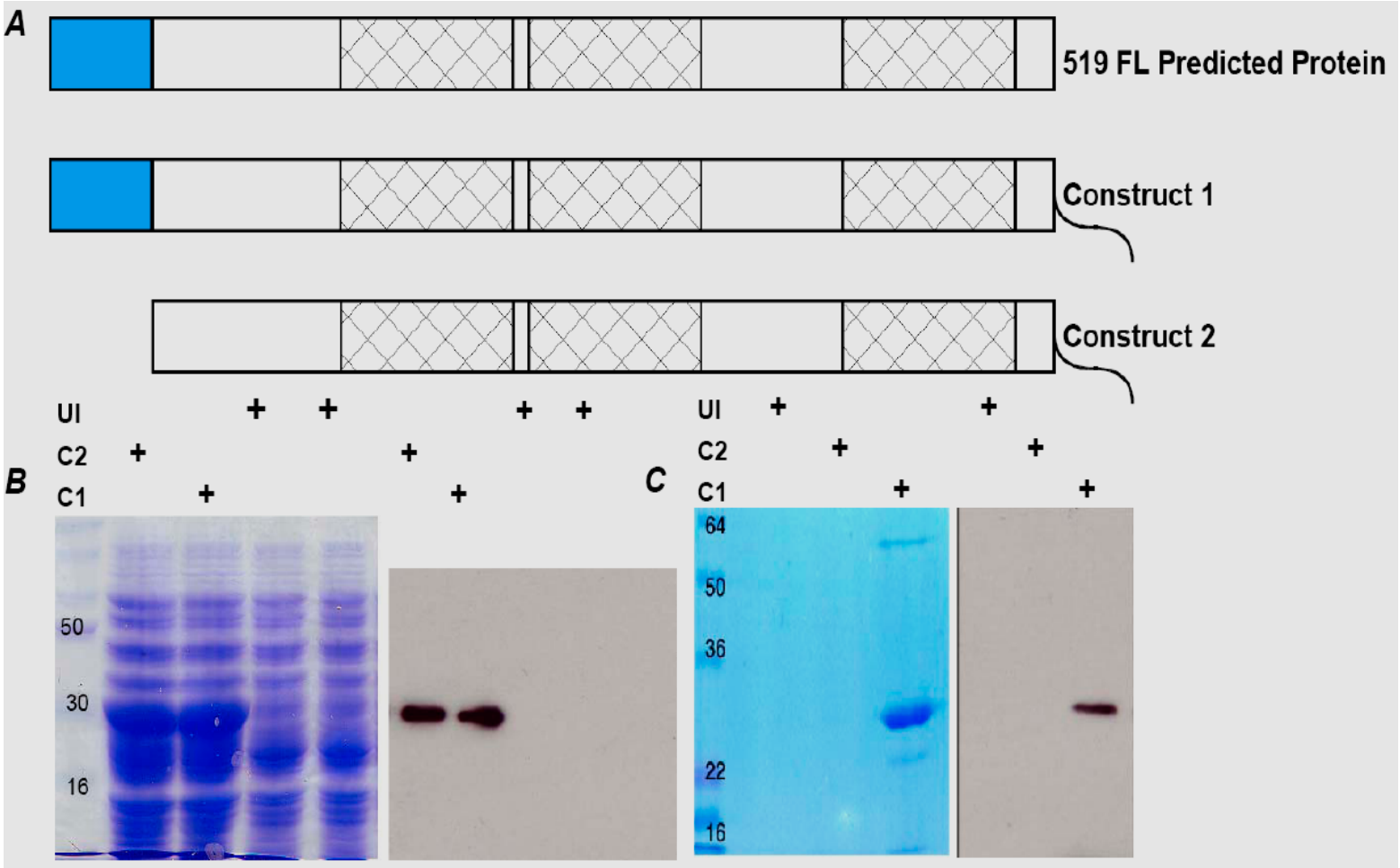
A
519 FL Predicted Protein
Construct 1
Construct 2
UI
C2
C1
B
50
30
16
C
64
50
36
22
16
