# Supplementary material for: Helicobacter pylori Evolution: Lineage- Specific Adaptations in Homologs of Eukaryotic Sel1-Like Genes
Source: PLoS Comput Biol. 2007 Aug 10;3(8):e151. doi: 10.1371/journal.pcbi.0030151 (PMC1941758; doi:10.1371/journal.pcbi.0030151)
Supplement: Supplementary file 3 [file pcbi.0030151.sg004.pdf]

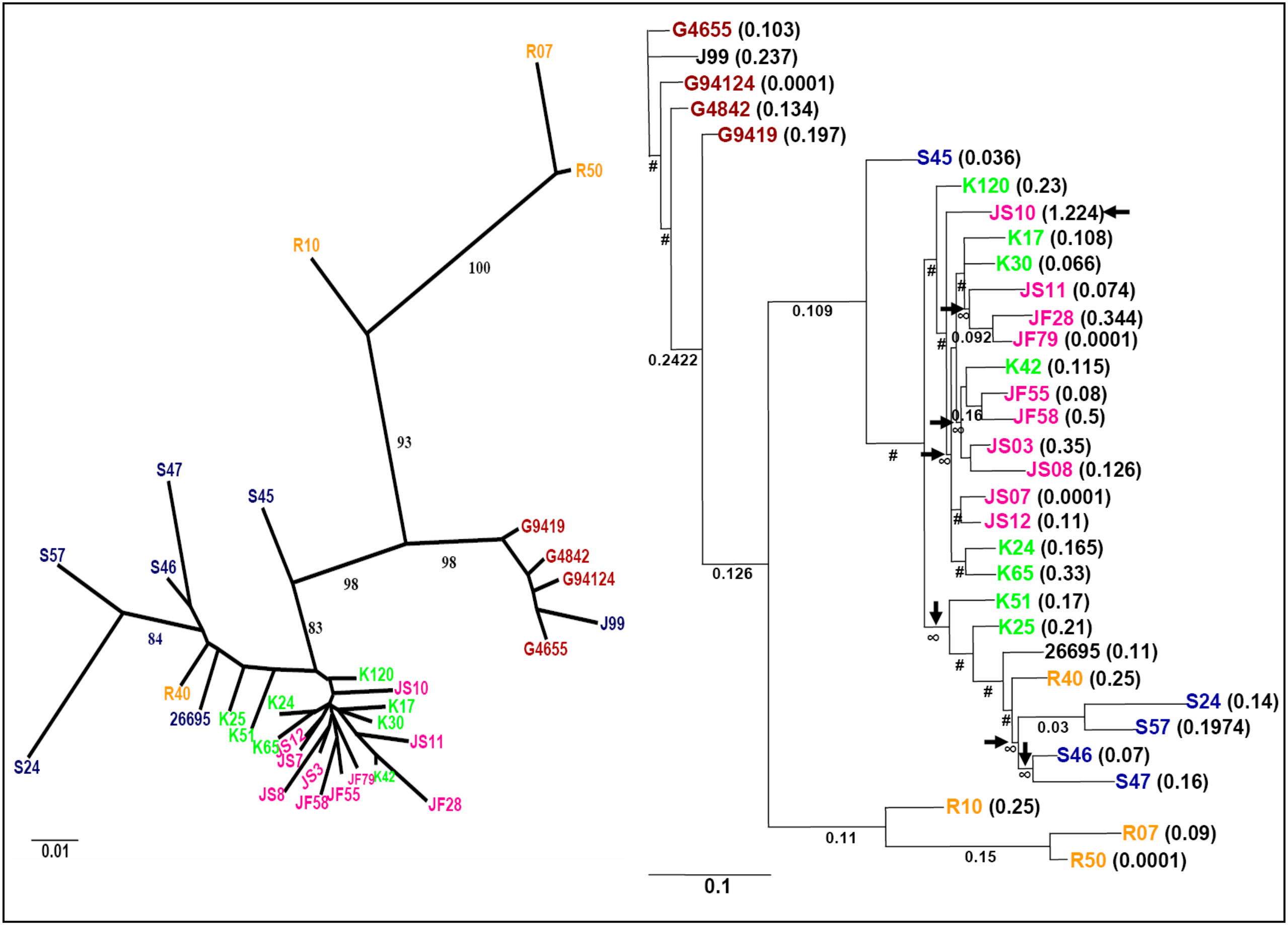

R07
R50
R10
100
93
S47
S45
G9419
S57
S46
G4842
98
98
G94124
84
83
J99
G4655
K120
R40
JS10
K24
K17
26695
K25
K30
K51
JS11
K65
JS12
JS7
JS3
JF79
K42
JS8
JF58
JF55
JF28
0.01
G4655 (0.103)
J99 (0.237)
G94124 (0.0001)
G4842 (0.134)
G9419 (0.197)
S45 (0.036)
K120 (0.23)
JS10 (1.224)
K17 (0.108)
K30 (0.066)
JS11 (0.074)
JF28 (0.344)
JF79 (0.0001)
0.092
K42 (0.115)
JF55 (0.08)
JF58 (0.5)
0.16
JS03 (0.35)
JS08 (0.126)
JS07 (0.0001)
JS12 (0.11)
K24 (0.165)
K65 (0.33)
K51 (0.17)
K25 (0.21)
26695 (0.11)
R40 (0.25)
S24 (0.14)
S57 (0.1974)
S46 (0.07)
S47 (0.16)
R10 (0.25)
R07 (0.09)
R50 (0.0001)
0.109
0.2422
0.126
0.03
0.11
0.15
0.1

A **B**
