# Supplementary material for: Helicobacter pylori Evolution: Lineage- Specific Adaptations in Homologs of Eukaryotic Sel1-Like Genes
Source: PLoS Comput Biol. 2007 Aug 10;3(8):e151. doi: 10.1371/journal.pcbi.0030151 (PMC1941758; doi:10.1371/journal.pcbi.0030151)
Supplement: Supplementary file 4 [file pcbi.0030151.sg005.pdf]

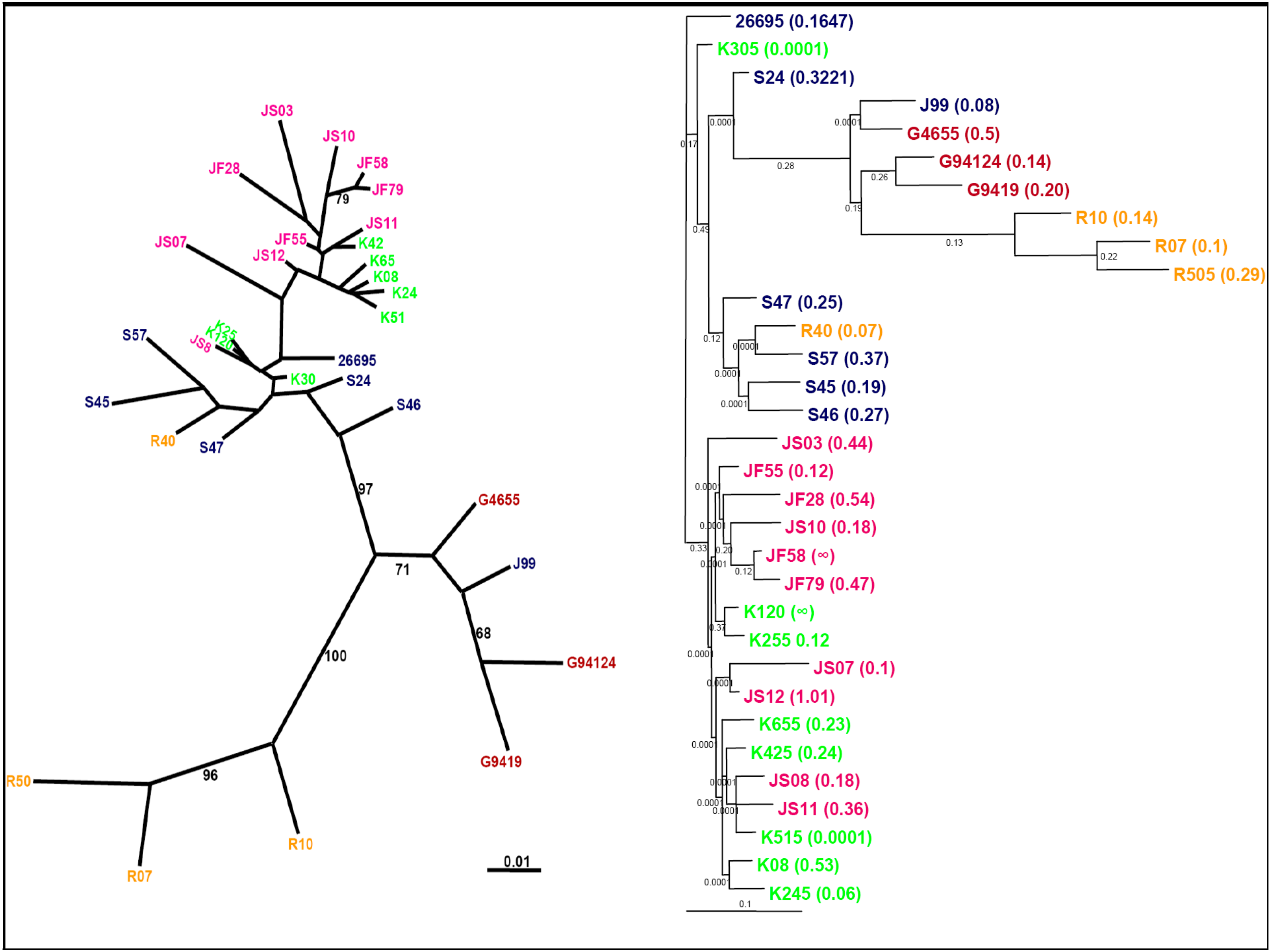
JS03
JS10
JF58
JF79
JF28
79
JS11
JF55
K42
JS07
JS12
K65
K08
K24
K51
S57
26695
K30
S24
S45
S46
R40
S47
97
G4655
J99
71
68
G94124
100
G9419
96
R50
R10
R07
0.01
26695 (0.1647)
K305 (0.0001)
S24 (0.3221)
J99 (0.08)
G4655 (0.5)
G94124 (0.14)
G9419 (0.20)
R10 (0.14)
R07 (0.1)
R505 (0.29)
S47 (0.25)
R40 (0.07)
S57 (0.37)
S45 (0.19)
S46 (0.27)
JS03 (0.44)
JF55 (0.12)
JF28 (0.54)
JS10 (0.18)
JF58 (∞)
JF79 (0.47)
K120 (∞)
K255 0.12
JS07 (0.1)
JS12 (1.01)
K655 (0.23)
K425 (0.24)
JS08 (0.18)
JS11 (0.36)
K515 (0.0001)
K08 (0.53)
K245 (0.06)
0.1

A

B
