# Supplementary material for: Helicobacter pylori Evolution: Lineage- Specific Adaptations in Homologs of Eukaryotic Sel1-Like Genes
Source: PLoS Comput Biol. 2007 Aug 10;3(8):e151. doi: 10.1371/journal.pcbi.0030151 (PMC1941758; doi:10.1371/journal.pcbi.0030151)
Supplement: Supplementary file 5 [file pcbi.0030151.sg006.pdf]

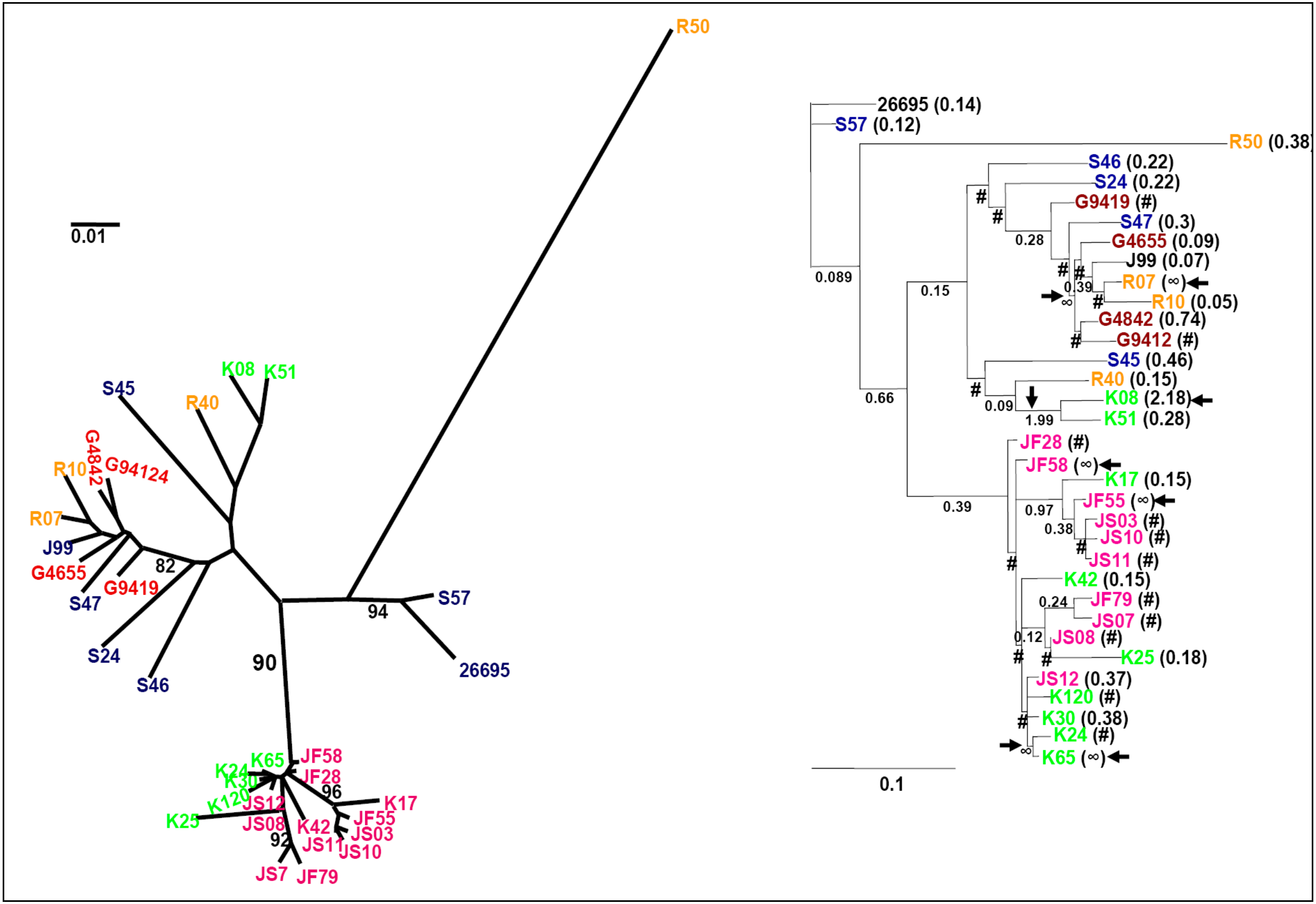

R50
0.01
S45
K08
K51
R40
G4842
G94124
R10
R07
J99
G4655
S47
G9419
82
S24
S46
90
94
S57
26695
K65
JF58
K24
K30
JF28
96
K17
K120
JS12
K25
JS08
K42
JF55
JS03
JS11
JS10
92
JS7
JF79
26695 (0.14)
S57 (0.12)
R50 (0.38)
S46 (0.22)
S24 (0.22)
G9419 (#)
S47 (0.3)
G4655 (0.09)
J99 (0.07)
R07 (∞)
R10 (0.05)
G4842 (0.74)
G9412 (#)
S45 (0.46)
R40 (0.15)
K08 (2.18)
K51 (0.28)
JF28 (#)
JF58 (∞)
K17 (0.15)
JF55 (∞)
JS03 (#)
JS10 (#)
JS11 (#)
K42 (0.15)
JF79 (#)
JS07 (#)
JS08 (#)
K25 (0.18)
JS12 (0.37)
K120 (#)
K30 (0.38)
K24 (#)
K65 (∞)
0.089
0.66
0.15
0.28
0.39
0.09
1.99
0.39
0.97
0.38
0.24
0.12
0.1

A B
