# Supplementary material for: Helicobacter pylori Evolution: Lineage- Specific Adaptations in Homologs of Eukaryotic Sel1-Like Genes
Source: PLoS Comput Biol. 2007 Aug 10;3(8):e151. doi: 10.1371/journal.pcbi.0030151 (PMC1941758; doi:10.1371/journal.pcbi.0030151)
Supplement: Supplementary file 6 [file pcbi.0030151.sg007.pdf]

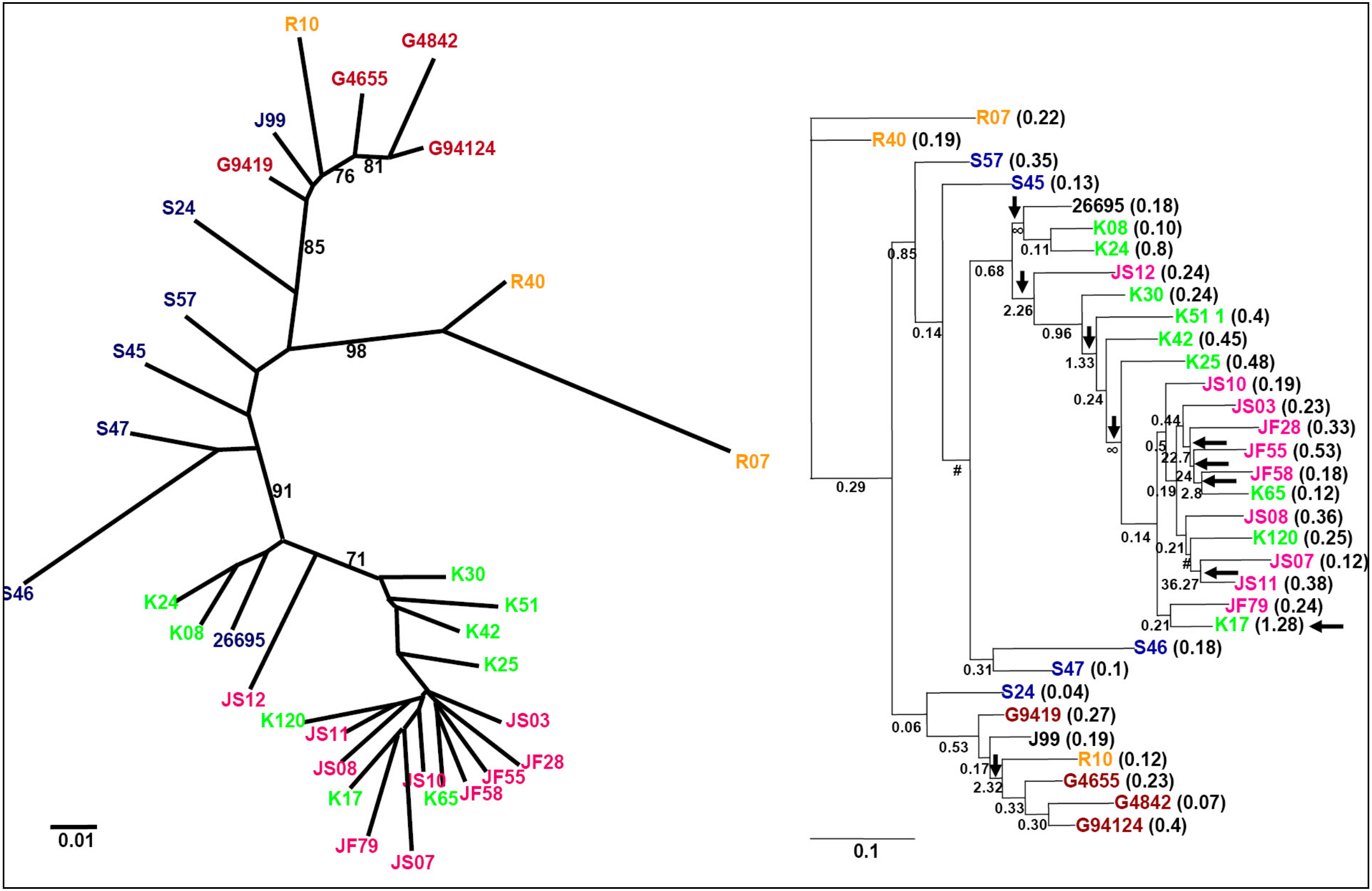
R10
G4842
G4655
J99
G94124
G9419
76
81
S24
85
R40
S57
98
S45
S47
91
R07
S46
71
K30
K24
K51
K08
26695
K42
K25
JS12
K120
JS03
JS11
JS08
JF28
JS10
JF55
K17
K65
JF58
JF79
JS07
0.01
R07 (0.22)
R40 (0.19)
S57 (0.35)
S45 (0.13)
26695 (0.18)
K08 (0.10)
K24 (0.8)
JS12 (0.24)
K30 (0.24)
K51 1 (0.4)
K42 (0.45)
K25 (0.48)
JS10 (0.19)
JS03 (0.23)
JF28 (0.33)
JF55 (0.53)
JF58 (0.18)
K65 (0.12)
JS08 (0.36)
K120 (0.25)
JS07 (0.12)
JS11 (0.38)
JF79 (0.24)
K17 (1.28)
S46 (0.18)
S47 (0.1)
S24 (0.04)
G9419 (0.27)
J99 (0.19)
R10 (0.12)
G4655 (0.23)
G4842 (0.07)
G94124 (0.4)
0.85
0.11
0.68
2.26
0.96
1.33
0.24
0.14
0.44
0.5
22.7
24
2.8
0.19
0.14
0.21
36.27
0.21
#
0.29
0.31
0.06
0.53
0.17
2.32
0.33
0.30
0.1

A **B**
