# Supplementary material for: Helicobacter pylori Evolution: Lineage- Specific Adaptations in Homologs of Eukaryotic Sel1-Like Genes
Source: PLoS Comput Biol. 2007 Aug 10;3(8):e151. doi: 10.1371/journal.pcbi.0030151 (PMC1941758; doi:10.1371/journal.pcbi.0030151)
Supplement: Supplementary file 7 [file pcbi.0030151.sg008.pdf]

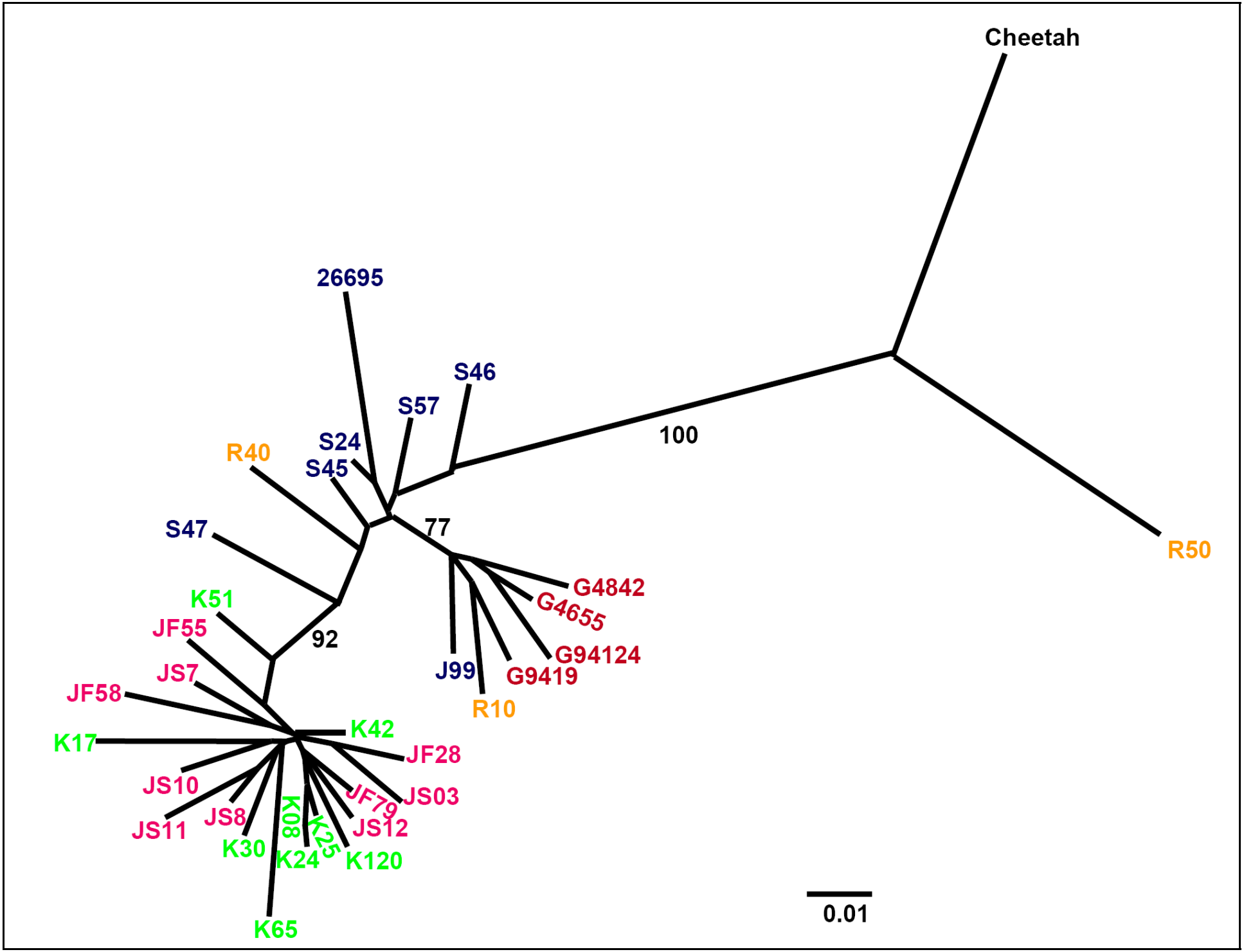

Cheetah
R50
100
26695
S46
S57
S24
S45
R40
S47
77
G4842
G4655
G94124
G9419
J99
R10
92
K51
JF55
JS7
JF58
K17
K42
JF28
JS03
JF79
JS12
K120
K25
K08
K24
K30
JS8
JS11
JS10
K65
0.01
