# Supplementary material for: Helicobacter pylori Evolution: Lineage- Specific Adaptations in Homologs of Eukaryotic Sel1-Like Genes
Source: PLoS Comput Biol. 2007 Aug 10;3(8):e151. doi: 10.1371/journal.pcbi.0030151 (PMC1941758; doi:10.1371/journal.pcbi.0030151)
Supplement: Supplementary file 8 [file pcbi.0030151.st006.pdf]

Table S6A. Selection pressures acting on Hp0519 : ML parameter estimates

Model Code	κappa	Tree Length	ln L [a]	dN / dS [b]	Estimates of Parameters	LRT [d.f.] [c]	χ^2	P	Positively Selected Sites (ω > 1); Reference Sequence 26695
Site-Specific Models									
M0 (one-ratio)	4.325	2.203	-4359.719	0.406	$\omega = 0.406$				None
M1 (neutral)	4.258	2.275	-4232.698	0.435	p0 = 0.564, $\omega_0 = 0$; p1= 0.435, $\omega_1 = 1$				Not Allowed
M2 (selection)	4.881	2.442	-4204.934	0.652	p0 = 0.553, p1 = 0.385, (p2 = 0.061), $\omega_2 = 4.408$	M0 vs M2 [2]<br>M1 vs M2 [2]	309.57<br>55.528	<0.000<br><0.000	44*, 81R, 82L, 87S, 114F, 117E, 132L, 136R, 171A, 189S, 216*, 220S, 221T, 230W, 264T, 269D, 279A, 290L
M3 (discrete)	4.613	2.458	-4199.613	0.541	p0 = 0.589, ω_0 = 0.023; p1 = 0.336, $\omega_1 = 0.796$; p2 = 0.073, $\omega_2 = 3.515$	M0 vs M3 [4]<br>M1 vs M3 [4]<br>M2 vs M3 [2]	320.21<br>66.17<br>10.642	<0.000<br><0.000<br><0.004	44*, 81R, 82L, 87S, 114F, 117E, 132L, 136R, 171A, 189S, 216*, 220S, 221T, 230W, 264T, 269D, 279A, 290L
M7 (β)	3.967	2.321	-4225.387	0.351	p = 0.093, q = 0.174				Not Allowed
M8 (β & ω)	4.619	2.461	-4199.561	0.542	p0 = 0.932, p = 0.116, q = 0.251, p2 = 0.067, $\omega_2 = 3.645$	M7 vs M8 [2]	51.652	<0.000	44*, 81R, 82L, 87S, 114F, 117E, 132L, 136R, 171A, 189S, 216*, 220S, 221T, 230W, 264T, 269D, 279A, 290L

a, InL, Log-likelihood score; b, dN/dS, rate ratio of non-sy nonymous to synonymous changes av eraged over all lineages and all s ites

c, likelihood ratio test [degrees of freedom];

Table S6B. Episodic adaptive evolution in the Japanese HP0519 lineage

Model Code	κappa	Tree Length	ln L [a]	Estimates of Parameters	LRT [d.f.] [b]	χ^2	P	Positively Selected Sites Foreground lineage (Reference Sequence 26695)	Positively Selected Sites Background lineage (Reference Sequence 26695)
A. Lineage-Specific Models [LSMs]									
FR (Free-Ratio)	4.344	2.191	-4313.6	one ω for each of 51 lineages	vs M0 [50]	92.2	<0.000	Not Applicable	Not Applicable
M2J (Two-ratio)	4.336	2.191	-4348.9	ω_1 (Background lineage) = 0.354 ; ω_j(Foreground lineage, Japan) = 1.581	vs M0 [1]	21.7	<0.000	Not Applicable	Not Applicable
B. Lineage-Site Specific Models [LSSMs]									
M2JM2	4.563	2.516	-4188.1	p0 = 0.551, $\omega_0 = 0$; p1 = 0.326, $\omega_1 = 1$; (p2 + p3) = 0.121, $\omega_j = 21.581$	vs M1 [2]	89.2	<0.000	39D, 40D, 44*, 56H, 82L , 153K, 162F, 166S, 171A, 181E, 182F, 184S, 185M, 186L, 209G , 210I, 213K , 219K, 221T, 222Q, 223I, 224K, 225D, 227T	Not Allowed
M2JM3	4.834	2.571	-4177.6	p0 = 0.636, ω_0 = 0.043; p1 = 0.242, $\omega_R = 1.497$; (p2 + p3) = 0.121, $\omega_j = 25.184$	vs M3 [2]	44.1	<0.000	39D, 40D, 44*, 56H, 82L , 153K, 162F, 166S, 171A, 181E, 182F, 184S , 185M , 186L, 209G , 210I , 212V , 213K , 219K, 221T, 222Q, 223I, 224K, 225D, 227T , 231E	12F, 14K, 15P, 16L, 21V, 25P, 33E, 38S, 54R, 79D, 81R, 87S, 102G, 107V , 114F, 122G , 131A, 132L, 136R , 140E, 144M, 157H, 161M, 173N, 176L, 177P, 178N, 180A, 183T, 190R, 199A, 206K, 220S, 226K, 229L, 230W, 241T, 267K, 269D, 270R, 279A

*, Sites listed in bold, >95% probability, sites in Italics, > 75% < 95%, sites in plain letters > 50% < 75% probability; sites listed in blue and red are those that are restricted to Region I and Region II of Hp0519 as shown in Figure 3b.

a, InL, log likelihood score; b, likelihood ratio test [degrees of freedom].

Table 6C: Selection pressures acting on Hp0519: ML parameter estimates assuming a 'Star" phylogeny

Model Code	ln L [a]	dN / dS [b]	Estimates of Parameters	LRT [d.f.] [c]	χ^2	P	Positively Selected Sites (ω > 1); Reference Sequence 26695
Site-Specific Models							
M0 (one-ratio)	-6320.0002	0.4816	$\omega = 0.481$				None
M1 (nearly neutral)	-5769.2927	0.313	p0 = 0.703, $\omega_0 = 0.02$; p1= 0.293, $\omega_1 = 1$				Not Allowed
M2 (selection)	-5610.7626	0.718	p0 = 0.661, $\omega_0 = 0.02$ p1 = 0.261, $\omega_1 = 1$ p2 = 0.061, $\omega_2 = 5.74$	M0 vs M2 [2]<br>M1 vs M2 [2]	1101.415<br>317.0602	<0.000<br><0.000	10L, 44*, 82L, 87S, 114F, 117E, 132 L, 136 R, 171 A, 176 L, 177 P, 182F, 220S, 221T, 222Q, 223 I, 267 K, 269 D, 288E, 290 L
M3 (discrete)	-5609.5942	0.766	p0 = 0.672 $\omega_0 = 0.023$; p1 = 0.256, $\omega_1 = 1.1$; p2 = 0.073, $\omega_2 = 6.34$	M0 vs M3 [4]<br>M1 vs M3 [4]	1420.81<br>319.39	<0.000<br><0.000	10L, 44*, 82L, 87S, 114F, 117E, 132 L, 136 R, 171 A, 176 L, 177 P, 182F, 220S, 221T, 222Q, 223 I, 267 K, 269 D, 288E, 290 L + 23 more sites (because of two ws under positive selection)
M7 (β)	-5757.4825	0.2955	p = 0.073 q = 0.174				Not Allowed
M8 (β & ω)	-5608.7399	0.64	p0 = 0.922, p = 0.068, q = 0.201, p1 = 0.077, $\omega_2 = 5.262$	M7 vs M8 [2]	297.48	<0.000	10L, 44*, 82L, 87S, 114F, 117E, 132 L, 136 R, 171 A, 176 L, 177 P, 182F, 220S, 221T, 222Q, 223 I, 267 K, 269 D, 288E, 290 L

See footnotes to Table 6a for explanation.
