# Supplementary material for: Helicobacter pylori Evolution: Lineage- Specific Adaptations in Homologs of Eukaryotic Sel1-Like Genes
Source: PLoS Comput Biol. 2007 Aug 10;3(8):e151. doi: 10.1371/journal.pcbi.0030151 (PMC1941758; doi:10.1371/journal.pcbi.0030151)
Supplement: Supplementary file 9 [file pcbi.0030151.st001.doc]

| **Table S1. Signal peptide prediction for  *H. pylori* SLR proteins** | | | | |
| --- | --- | --- | --- | --- |
| Predicted TPR protein | Signal Prediction (Using Gram -ve Neural Networks) | Signal Predict Probability (by HMM) | Predicted Cleavage Site | Probability of Cleavage Site (%) |
| Hp0211 | Yes | 98.6 | 25-26 / LMA-EP | 85 |
| Hp0235 | Yes | 67.3 | 22-23 / AHL-WG | 45 |
| Hp0336 | No (non-secretory) | 0 | - | 0 |
| Hp0628* | No (non-secretory) | 0 | - | 0 |
| Hp0160 | Yes | 100 | 25-26 / LVA-TT | 90.2 |
| Hp0519 | Yes | 99.9 | 31-32 / CIA-DE | 99.9 |
| Hp1117 | Yes | 97.9 | 23-24 / ALG-AE | 90.3 |
| Hp1098 | Yes | 99.4 | 25-26 / LMA-EQ | 82 |
| Jhp0148 | Yes | 100 | 25-26 / LMA-EP | 81.2 |
| Jhp0197 | Yes | 99.5 | 26-27 / LMA-EP | 86 |
| Jhp0220 | Yes | 96.6 | 22-23 / AHL-WG | 52 |
| Jhp0318 | No (non-secretory) | 0 | - | 0 |
| Jhp0468 | Yes | 99.1 | 31-32 / CIA-DE | 99 |
| Jhp0571* | Yes | 99.9 | 19-20 / SGA-EE | 73 |
| Jhp1024 | Yes | 99.3 | 25-26 / LMA-EQ | 84 |
| Jhp1045 | No (non-secretory) | 0 | - | 0 |
| Jhp1437 | Yes | 100 | 26-27 / LMA-EQ | 82 |
| *, *jhp0571* homolog in strain 26695 is split into two ORFs (*hp0627* and *hp0628*) | | | | |
