# Supplementary material for: Helicobacter pylori Evolution: Lineage- Specific Adaptations in Homologs of Eukaryotic Sel1-Like Genes
Source: PLoS Comput Biol. 2007 Aug 10;3(8):e151. doi: 10.1371/journal.pcbi.0030151 (PMC1941758; doi:10.1371/journal.pcbi.0030151)
Supplement: Supplementary file 10 [file pcbi.0030151.st002.doc]

| **Table S2. Confidently predicted domains, repeats, motifs and features using SMART (Simple Modular Architecture Research Tool; http://smart.embl-heidelberg.de/)** | | | | | | | | |
| --- | --- | --- | --- | --- | --- | --- | --- | --- |
| **Hp-0235** |  |  |  |  | **Jhp0220** |  |  |  |
| **name** | **begin** | **end** | **E-value** |  | **name** | **begin** | **end** | **E-value** |
| SEL1 | 59 | 94 | 6.70E-10 |  | [SEL1](http://smart.embl-heidelberg.de/smart/do_annotation.pl?DOMAIN=SEL1&TYPE=SMART&START=59&END=94&LENGTH=35&E_VALUE=6.7e-10&BLAST=EGCTQLGIIYENGQGTRIDYKKALEYYKTACQADD) | 59 | 94 | 6.70E-10 |
| [SEL1](http://smart.embl-heidelberg.de/smart/do_annotation.pl?DOMAIN=SEL1&TYPE=SMART&START=95&END=130&LENGTH=35&E_VALUE=9.3e-07&BLAST=EGCFGLGGLYDEGLGTTQNYQEAIDAYAKACVLKH) | 95 | 130 | 9.30E-07 |  | [SEL1](http://smart.embl-heidelberg.de/smart/do_annotation.pl?DOMAIN=SEL1&TYPE=SMART&START=95&END=130&LENGTH=35&E_VALUE=2e-06&BLAST=EGCFGLGGLYDEGLGTAQNYQEAIDAYAKACVLKH) | 95 | 130 | 2.00E-06 |
| [SEL1](http://smart.embl-heidelberg.de/smart/do_annotation.pl?DOMAIN=SEL1&TYPE=SMART&START=131&END=164&LENGTH=33&E_VALUE=0.00083&BLAST=ESCYNLGIIYDRKIKGNADQAVTYYQKSCNFDM) | 131 | 164 | 8.30E-04 |  | [SEL1](http://smart.embl-heidelberg.de/smart/do_annotation.pl?DOMAIN=SEL1&TYPE=SMART&START=131&END=164&LENGTH=33&E_VALUE=0.00076&BLAST=ESCYNLGIIYDRKIKGNAAQAVTYYQKSCNFDM) | 131 | 164 | 7.60E-04 |
| [SEL1](http://smart.embl-heidelberg.de/smart/do_annotation.pl?DOMAIN=SEL1&TYPE=SMART&START=165&END=201&LENGTH=36&E_VALUE=3.5e-07&BLAST=KGCYVLGVAYEKGFLEVKQSNHKAVIYYLKACRLDD) | 165 | 201 | 3.50E-07 |  | [SEL1](http://smart.embl-heidelberg.de/smart/do_annotation.pl?DOMAIN=SEL1&TYPE=SMART&START=165&END=201&LENGTH=36&E_VALUE=8.3e-07&BLAST=KGCYILGTAYEKGFLEVKQSNHKAVIYYLKACRLNE) | 165 | 201 | 8.30E-07 |
| [SEL1](http://smart.embl-heidelberg.de/smart/do_annotation.pl?DOMAIN=SEL1&TYPE=SMART&START=202&END=238&LENGTH=36&E_VALUE=5.4e-05&BLAST=QACRALGSLFENGDAGLDEDFEVAFDYLQKACGLNN) | 202 | 238 | 5.40E-05 |  | [SEL1](http://smart.embl-heidelberg.de/smart/do_annotation.pl?DOMAIN=SEL1&TYPE=SMART&START=202&END=238&LENGTH=36&E_VALUE=1.7e-05&BLAST=QACRALGSLFENGDAGLDEDFEVAFDYLQKACALNN) | 202 | 238 | 1.70E-05 |
| [SEL1](http://smart.embl-heidelberg.de/smart/do_annotation.pl?DOMAIN=SEL1&TYPE=SMART&START=239&END=274&LENGTH=35&E_VALUE=2.8e-09&BLAST=GGCASLGSMYMLGRYVKKDPQKAFNFFKQACDMGS) | 239 | 274 | 2.80E-09 |  | [SEL1](http://smart.embl-heidelberg.de/smart/do_annotation.pl?DOMAIN=SEL1&TYPE=SMART&START=239&END=274&LENGTH=35&E_VALUE=5.4e-10&BLAST=GGCASLGSMYMLGRYVKKDPQKAFNYFKQACDMGS) | 239 | 274 | 5.40E-10 |
| [SEL1](http://smart.embl-heidelberg.de/smart/do_annotation.pl?DOMAIN=SEL1&TYPE=SMART&START=275&END=310&LENGTH=35&E_VALUE=3.3e-08&BLAST=VSCSRMGFMYSQGDAVPKDLRKALDNYERGCDMGD) | 275 | 310 | 3.30E-08 |  | [SEL1](http://smart.embl-heidelberg.de/smart/do_annotation.pl?DOMAIN=SEL1&TYPE=SMART&START=275&END=310&LENGTH=35&E_VALUE=1.1e-07&BLAST=VSCSRMGFMYSQGDTVSKDLRKALDNYERGCDMGD) | 275 | 310 | 1.10E-07 |
| [SEL1](http://smart.embl-heidelberg.de/smart/do_annotation.pl?DOMAIN=SEL1&TYPE=SMART&START=311&END=342&LENGTH=31&E_VALUE=0.44&BLAST=VGCFALAGMYYNMKDKENAIMIYDKGCKLGM) | 311 | 342 | 4.40E-01 |  | [SEL1](http://smart.embl-heidelberg.de/smart/do_annotation.pl?DOMAIN=SEL1&TYPE=SMART&START=311&END=342&LENGTH=31&E_VALUE=0.44&BLAST=VGCFALAGMYYNMKDKENAIMIYDKGCKLGM) | 311 | 342 | 4.40E-01 |
|  |  |  |  |  |  |  |  |  |
| **Hp-0628** |  |  |  |  | **Jhp-0571** |  |  |  |
| **name** | **begin** | **end** | **E-value** |  | **name** | **begin** | **end** | **E-value** |
| [SEL1](http://smart.embl-heidelberg.de/smart/do_annotation.pl?DOMAIN=SEL1&TYPE=SMART&START=8&END=44&LENGTH=36&E_VALUE=0.00075&BLAST=TACANLAQMYENKKNADSNDKENALQLYAVACQGGD) | 8 | 44 | 7.50E-04 |  | [Pfam:Brevenin](http://smart.embl-heidelberg.de/smart/do_annotation.pl?DOMAIN=Brevenin&TYPE=Pfam&START=1&END=43&LENGTH=42&E_VALUE=4.9&BLAST=LKKSLLLLVFLILQLSGAEENNQAPKNTPPELNPANTKGAPN) | 1 | 43 | 4.90E+00 |
| [SEL1](http://smart.embl-heidelberg.de/smart/do_annotation.pl?DOMAIN=SEL1&TYPE=SMART&START=45&END=80&LENGTH=35&E_VALUE=1e-11&BLAST=LACNNLGWMFANGSGVPKDYYKAISYYKFSCENGN) | 45 | 80 | 1.00E-11 |  | [SEL1](http://smart.embl-heidelberg.de/smart/do_annotation.pl?DOMAIN=SEL1&TYPE=SMART&START=67&END=98&LENGTH=31&E_VALUE=23&BLAST=QTELSAGIDLAKKGDYQGAFKLFSQSCDNGN) | 67 | 98 | 2.30E+01 |
| [SEL1](http://smart.embl-heidelberg.de/smart/do_annotation.pl?DOMAIN=SEL1&TYPE=SMART&START=119&END=155&LENGTH=36&E_VALUE=5.4e-06&BLAST=MGCANLGWIYANGDLGAPLNNHYAAKYFQMACDGGI) | 119 | 155 | 5.40E-06 |  | [SEL1](http://smart.embl-heidelberg.de/smart/do_annotation.pl?DOMAIN=SEL1&TYPE=SMART&START=99&END=134&LENGTH=35&E_VALUE=6.7e-06&BLAST=AGCFAVGAMYANGVGIQTNRLKAARYYEMGCSGGD) | 99 | 134 | 6.70E-06 |
| [SEL1](http://smart.embl-heidelberg.de/smart/do_annotation.pl?DOMAIN=SEL1&TYPE=SMART&START=156&END=191&LENGTH=35&E_VALUE=1.1e-08&BLAST=GSCNNLGVLYQKGLGVPQDDQRALDLFSYACDNGF) | 156 | 191 | 1.10E-08 |  | [SEL1](http://smart.embl-heidelberg.de/smart/do_annotation.pl?DOMAIN=SEL1&TYPE=SMART&START=135&END=171&LENGTH=36&E_VALUE=0.00075&BLAST=TACANLAQMYENKKNADSNDKENALQLYAVACQGGD) | 135 | 171 | 7.50E-04 |
|  |  |  |  |  | [SEL1](http://smart.embl-heidelberg.de/smart/do_annotation.pl?DOMAIN=SEL1&TYPE=SMART&START=172&END=207&LENGTH=35&E_VALUE=8.6e-12&BLAST=LACNNLGWMFANGSGVPKDYYKAMSYYKFSCENGN) | 172 | 207 | 8.60E-12 |
|  |  |  |  |  | [SEL1](http://smart.embl-heidelberg.de/smart/do_annotation.pl?DOMAIN=SEL1&TYPE=SMART&START=246&END=282&LENGTH=36&E_VALUE=5.4e-06&BLAST=MGCANLGWIYANGDLGAPLNNHYAAKYFQMACDGGI) | 246 | 282 | 5.40E-06 |
|  |  |  |  |  | [SEL1](http://smart.embl-heidelberg.de/smart/do_annotation.pl?DOMAIN=SEL1&TYPE=SMART&START=283&END=318&LENGTH=35&E_VALUE=1.1e-08&BLAST=GSCNNLGVLYQKGLGVPQDDQRALDLFSYACDNGF) | 283 | 318 | 1.10E-08 |
|  |  |  |  |  |  |  |  |  |
| **Hp-0160** |  |  |  |  | **Jhp-0148** |  |  |  |
| **name** | **begin** | **end** | **E-value** |  | **name** | **begin** | **end** | **E-value** |
| [SEL1](http://smart.embl-heidelberg.de/smart/do_annotation.pl?DOMAIN=SEL1&TYPE=SMART&START=28&END=59&LENGTH=31&E_VALUE=67&BLAST=EKYFKMATQAFKRGDYHKAVAFYKRSCNLRV) | 28 | 59 | 6.70E+01 |  | [SEL1](http://smart.embl-heidelberg.de/smart/do_annotation.pl?DOMAIN=SEL1&TYPE=SMART&START=28&END=59&LENGTH=31&E_VALUE=77&BLAST=EKYFKMANQALKRGDYHRAVAFYKRSCNLRM) | 28 | 59 | 7.70E+01 |
| [SEL1](http://smart.embl-heidelberg.de/smart/do_annotation.pl?DOMAIN=SEL1&TYPE=SMART&START=60&END=95&LENGTH=35&E_VALUE=1.2e-07&BLAST=VGCTSLGSMYEDGDGVDQNITKAVFYYRRGCNLRN) | 60 | 95 | 1.20E-07 |  | [SEL1](http://smart.embl-heidelberg.de/smart/do_annotation.pl?DOMAIN=SEL1&TYPE=SMART&START=60&END=95&LENGTH=35&E_VALUE=2.2e-08&BLAST=VGCTSLGSMYEYGDGVDQNISKAVFYYRRGCNLRN) | 60 | 95 | 2.20E-08 |
| [SEL1](http://smart.embl-heidelberg.de/smart/do_annotation.pl?DOMAIN=SEL1&TYPE=SMART&START=96&END=131&LENGTH=35&E_VALUE=1.6e-07&BLAST=LACASLGSMYEDGDGVQKNLPKAIYYYRRGCHLKG) | 96 | 131 | 1.60E-07 |  | [SEL1](http://smart.embl-heidelberg.de/smart/do_annotation.pl?DOMAIN=SEL1&TYPE=SMART&START=96&END=131&LENGTH=35&E_VALUE=9.2e-08&BLAST=LACASLGSMYEDGDGVQKDFPKAIYYYRRGCHLKG) | 96 | 131 | 9.20E-08 |
| [SEL1](http://smart.embl-heidelberg.de/smart/do_annotation.pl?DOMAIN=SEL1&TYPE=SMART&START=132&END=167&LENGTH=35&E_VALUE=1.7e-07&BLAST=VSCGSLGFMYFNGTGVKQNYAKALFLSKYACSLNY) | 132 | 167 | 1.70E-07 |  | [SEL1](http://smart.embl-heidelberg.de/smart/do_annotation.pl?DOMAIN=SEL1&TYPE=SMART&START=132&END=167&LENGTH=35&E_VALUE=2e-07&BLAST=VSCGSLGFMYFNGTGVKQNYAKALSFSKYACSLNY) | 132 | 167 | 2.00E-07 |
| [SEL1](http://smart.embl-heidelberg.de/smart/do_annotation.pl?DOMAIN=SEL1&TYPE=SMART&START=168&END=203&LENGTH=35&E_VALUE=1e-05&BLAST=ISCNFVGYMYRNAKGVQKDLKKALANFKRGCHLKD) | 168 | 203 | 1.00E-05 |  | [SEL1](http://smart.embl-heidelberg.de/smart/do_annotation.pl?DOMAIN=SEL1&TYPE=SMART&START=168&END=203&LENGTH=35&E_VALUE=5.1e-05&BLAST=ISCNFVGYMYKSAKGVEKDLKKALANFKRGCHLKD) | 168 | 203 | 5.10E-05 |
| [SEL1](http://smart.embl-heidelberg.de/smart/do_annotation.pl?DOMAIN=SEL1&TYPE=SMART&START=204&END=239&LENGTH=35&E_VALUE=1.2e-05&BLAST=ASCVSLGYMYEVGMDVKQNGEQALNLYKKGCYLKR) | 204 | 239 | 1.20E-05 |  | [SEL1](http://smart.embl-heidelberg.de/smart/do_annotation.pl?DOMAIN=SEL1&TYPE=SMART&START=204&END=239&LENGTH=35&E_VALUE=2e-06&BLAST=ASCVSLGYLYEAGMDVKQNEEQALNLYKKGCSLKE) | 204 | 239 | 2.00E-06 |
| [SEL1](http://smart.embl-heidelberg.de/smart/do_annotation.pl?DOMAIN=SEL1&TYPE=SMART&START=240&END=275&LENGTH=35&E_VALUE=5.6e-10&BLAST=SGCHNVAVMYYTGKGVPKDLDKAISYYKKGCTLGF) | 240 | 275 | 5.60E-10 |  | [SEL1](http://smart.embl-heidelberg.de/smart/do_annotation.pl?DOMAIN=SEL1&TYPE=SMART&START=240&END=275&LENGTH=35&E_VALUE=9.8e-09&BLAST=SGCHNVAVMYYTGKGAPKDLEKATSYYKKGCALGF) | 240 | 275 | 9.80E-09 |
|  |  |  |  |  |  |  |  |  |
| **Hp-0211** |  |  |  |  | **Jhp-0197** |  |  |  |
| **name** | **begin** | **end** | **E-value** |  | **name** | **begin** | **end** | **E-value** |
| [signal peptide](http://smart.embl-heidelberg.de/smart/show_segment.pl?NAME=SIGNAL&START=1&END=26&BLAST=LGNVKKTLFGVLCLGTLCLRGLMAE) | 1 | 26 | - |  | [signal peptide](http://smart.embl-heidelberg.de/smart/show_segment.pl?NAME=SIGNAL&START=1&END=26&BLAST=LGSVKKTLFGVLCLGALCLRGLMAE) | 1 | 26 | - |
| [SEL1](http://smart.embl-heidelberg.de/smart/do_annotation.pl?DOMAIN=SEL1&TYPE=SMART&START=29&END=60&LENGTH=31&E_VALUE=93&BLAST=KELVNLGIESAKKQDFAQAKTHFEKACELKN) | 29 | 60 | 9.30E+01 |  | [SEL1](http://smart.embl-heidelberg.de/smart/do_annotation.pl?DOMAIN=SEL1&TYPE=SMART&START=27&END=60&LENGTH=33&E_VALUE=220&BLAST=DAKELVSLGIESVKKQDFAQAKAHFEKACELKE) | 27 | 60 | 2.20E+02 |
| [SEL1](http://smart.embl-heidelberg.de/smart/do_annotation.pl?DOMAIN=SEL1&TYPE=SMART&START=61&END=96&LENGTH=35&E_VALUE=1.4e-08&BLAST=FGCVFLGAFYEEGKGVGKDLKKAIQFYTKGCELND) | 61 | 96 | 1.40E-08 |  | [SEL1](http://smart.embl-heidelberg.de/smart/do_annotation.pl?DOMAIN=SEL1&TYPE=SMART&START=61&END=96&LENGTH=35&E_VALUE=1.4e-08&BLAST=FGCVFLGAFYEEGKGVGKDLKKAIQFYTKGCELND) | 61 | 96 | 1.40E-08 |
| [SEL1](http://smart.embl-heidelberg.de/smart/do_annotation.pl?DOMAIN=SEL1&TYPE=SMART&START=97&END=132&LENGTH=35&E_VALUE=5.4e-10&BLAST=YGCNLLGNLYYNGQGVSKDAKKASQYYSKACDLNH) | 97 | 132 | 5.40E-10 |  | [SEL1](http://smart.embl-heidelberg.de/smart/do_annotation.pl?DOMAIN=SEL1&TYPE=SMART&START=97&END=132&LENGTH=35&E_VALUE=4.5e-09&BLAST=YGCRLLGNLYYNGQGVSKDAKKASQYYSKSCELNH) | 97 | 132 | 4.50E-09 |
| [SEL1](http://smart.embl-heidelberg.de/smart/do_annotation.pl?DOMAIN=SEL1&TYPE=SMART&START=133&END=168&LENGTH=35&E_VALUE=6.9e-10&BLAST=EGCMVLGSLHHYGVGTPKDLRKALDLYEKACDLKD) | 133 | 168 | 6.90E-10 |  | [SEL1](http://smart.embl-heidelberg.de/smart/do_annotation.pl?DOMAIN=SEL1&TYPE=SMART&START=133&END=168&LENGTH=35&E_VALUE=1.8e-09&BLAST=EGCTVLGSLHHYGVGTPKDLRKALDLYEKACDLKD) | 133 | 168 | 1.80E-09 |
| [SEL1](http://smart.embl-heidelberg.de/smart/do_annotation.pl?DOMAIN=SEL1&TYPE=SMART&START=169&END=200&LENGTH=31&E_VALUE=1.7&BLAST=PGCINAGYIYSVTKNFKEAIVRYSKACELKD) | 169 | 200 | 1.70E+00 |  | [SEL1](http://smart.embl-heidelberg.de/smart/do_annotation.pl?DOMAIN=SEL1&TYPE=SMART&START=169&END=200&LENGTH=31&E_VALUE=0.21&BLAST=PGCINAGYMYGVAKNFKEAIVRYSKACELKD) | 169 | 200 | 2.10E-01 |
| [SEL1](http://smart.embl-heidelberg.de/smart/do_annotation.pl?DOMAIN=SEL1&TYPE=SMART&START=201&END=236&LENGTH=35&E_VALUE=4.3e-06&BLAST=RGCYNLGVMQYNAQGTAKDEKQAVENFKKGCKSSV) | 201 | 236 | 4.30E-06 |  | [SEL1](http://smart.embl-heidelberg.de/smart/do_annotation.pl?DOMAIN=SEL1&TYPE=SMART&START=201&END=236&LENGTH=35&E_VALUE=4.3e-06&BLAST=RGCYNLGVMQYNAQGTAKDEKQAVENFKKGCKSSV) | 201 | 236 | 4.30E-06 |
|  |  |  |  |  |  |  |  |  |
| **Hp-0519** |  |  |  |  | **Jhp-468** |  |  |  |
| **name** | **begin** | **end** | **E-value** |  | **name** | **begin** | **end** | **E-value** |
| [low complexity](http://smart.embl-heidelberg.de/smart/show_segment.pl?NAME=SEG&START=10&END=28&BLAST=SFLKPLLLLLVLLLPFSL) | 10 | 28 | - |  | [low complexity](http://smart.embl-heidelberg.de/smart/show_segment.pl?NAME=SEG&START=10&END=28&BLAST=SFLRSLLLLLGLLLPFSL) | 10 | 28 | - |
| [SEL1](http://smart.embl-heidelberg.de/smart/do_annotation.pl?DOMAIN=SEL1&TYPE=SMART&START=81&END=115&LENGTH=34&E_VALUE=0.0068&BLAST=LGSVYLAKMYLEGKGVKVDYKKAQFYAENAIKGY) | 81 | 115 | 6.80E-03 |  | [SEL1](http://smart.embl-heidelberg.de/smart/do_annotation.pl?DOMAIN=SEL1&TYPE=SMART&START=89&END=123&LENGTH=34&E_VALUE=0.0057&BLAST=LGNVYLAKMYLEGKGVKVDYKKAQFYAQNAIKGY) | 89 | 123 | 5.70E-03 |
| [SEL1](http://smart.embl-heidelberg.de/smart/do_annotation.pl?DOMAIN=SEL1&TYPE=SMART&START=120&END=155&LENGTH=35&E_VALUE=0.55&BLAST=GGALILGRMQAEGLGMKKDLKQALKTYRHVVRMFS) | 120 | 155 | 5.50E-01 |  | [SEL1](http://smart.embl-heidelberg.de/smart/do_annotation.pl?DOMAIN=SEL1&TYPE=SMART&START=128&END=163&LENGTH=35&E_VALUE=0.55&BLAST=GGALILGRMQAEGLGMKKDLKQALKTYRHVVRMFS) | 128 | 163 | 5.50E-01 |
| [SEL1](http://smart.embl-heidelberg.de/smart/do_annotation.pl?DOMAIN=SEL1&TYPE=SMART&START=234&END=269&LENGTH=35&E_VALUE=0.0031&BLAST=EILYRIGIAYKEGLGTRKKKDRAKKFLQKSAEFGY) | 234 | 269 | 3.10E-03 |  | [SEL1](http://smart.embl-heidelberg.de/smart/do_annotation.pl?DOMAIN=SEL1&TYPE=SMART&START=241&END=276&LENGTH=35&E_VALUE=0.0031&BLAST=EILYRIGIAYKEGLGTRKKKDRAKKFLQKSAEFGY) | 241 | 276 | 3.10E-03 |
|  |  |  |  |  |  |  |  |  |
| **Hp-1098** |  |  |  |  | **Jhp-1024** |  |  |  |
| **name** | **begin** | **end** | **E-value** |  | **name** | **begin** | **end** | **E-value** |
| [low complexity](http://smart.embl-heidelberg.de/smart/show_segment.pl?NAME=SEG&START=13&END=23&BLAST=CLGALCLGGL) | 13 | 23 | - |  | [low complexity](http://smart.embl-heidelberg.de/smart/show_segment.pl?NAME=SEG&START=13&END=23&BLAST=CLGALCLGGL) | 13 | 23 | - |
| [SEL1](http://smart.embl-heidelberg.de/smart/do_annotation.pl?DOMAIN=SEL1&TYPE=SMART&START=29&END=60&LENGTH=31&E_VALUE=28&BLAST=KELVGLGAKSYKEKDFTQAKKYFEKACDLKE) | 29 | 60 | 2.80E+01 |  | [SEL1](http://smart.embl-heidelberg.de/smart/do_annotation.pl?DOMAIN=SEL1&TYPE=SMART&START=29&END=60&LENGTH=31&E_VALUE=30&BLAST=KELVGLGAKSYKEQDFTQAKKYFEKACDLKE) | 29 | 60 | 3.00E+01 |
| [SEL1](http://smart.embl-heidelberg.de/smart/do_annotation.pl?DOMAIN=SEL1&TYPE=SMART&START=61&END=96&LENGTH=35&E_VALUE=3.6e-09&BLAST=SGCFNLGVLYYQGQGVEKNLKKAASFYAKACDLNY) | 61 | 96 | 3.60E-09 |  | [SEL1](http://smart.embl-heidelberg.de/smart/do_annotation.pl?DOMAIN=SEL1&TYPE=SMART&START=61&END=96&LENGTH=35&E_VALUE=1.4e-09&BLAST=SGCFNLGVLYYQGHGVEKNLKKAASFYSKACDLNY) | 61 | 96 | 1.40E-09 |
| [SEL1](http://smart.embl-heidelberg.de/smart/do_annotation.pl?DOMAIN=SEL1&TYPE=SMART&START=97&END=132&LENGTH=35&E_VALUE=1.1e-09&BLAST=NGCHLLGNLYYSGQGVSQNTNKALQYYSKACDLKY) | 97 | 132 | 1.10E-09 |  | [SEL1](http://smart.embl-heidelberg.de/smart/do_annotation.pl?DOMAIN=SEL1&TYPE=SMART&START=97&END=132&LENGTH=35&E_VALUE=1.1e-09&BLAST=NGCHLLGNLYYSGQGVSQNTNKALQYYSKACDLKY) | 97 | 132 | 1.10E-09 |
| [SEL1](http://smart.embl-heidelberg.de/smart/do_annotation.pl?DOMAIN=SEL1&TYPE=SMART&START=133&END=168&LENGTH=35&E_VALUE=9.7e-10&BLAST=EGCASLGGIYHDGKVVTRDFKKAVEYFTKACDLND) | 133 | 168 | 9.70E-10 |  | [SEL1](http://smart.embl-heidelberg.de/smart/do_annotation.pl?DOMAIN=SEL1&TYPE=SMART&START=133&END=168&LENGTH=35&E_VALUE=9.7e-10&BLAST=EGCASLGGIYHDGKVVTRDFKKAVEYFTKACDLND) | 133 | 168 | 9.70E-10 |
| [SEL1](http://smart.embl-heidelberg.de/smart/do_annotation.pl?DOMAIN=SEL1&TYPE=SMART&START=169&END=204&LENGTH=35&E_VALUE=2.2e-07&BLAST=DGCTILGSLYDAGRGTPKDLKKALASYDKACDLKD) | 169 | 204 | 2.20E-07 |  | [SEL1](http://smart.embl-heidelberg.de/smart/do_annotation.pl?DOMAIN=SEL1&TYPE=SMART&START=169&END=204&LENGTH=35&E_VALUE=2.2e-07&BLAST=DGCTILGSLYDAGRGTPKDLKKALASYDKACDLKD) | 169 | 204 | 2.20E-07 |
| [SEL1](http://smart.embl-heidelberg.de/smart/do_annotation.pl?DOMAIN=SEL1&TYPE=SMART&START=205&END=240&LENGTH=35&E_VALUE=2.4e-07&BLAST=PGCFNAGNMYHHGEGATKNFKEALARYSKACELEN) | 205 | 240 | 2.40E-07 |  | [SEL1](http://smart.embl-heidelberg.de/smart/do_annotation.pl?DOMAIN=SEL1&TYPE=SMART&START=205&END=240&LENGTH=35&E_VALUE=3.9e-07&BLAST=PGCFNAGNMYHHGEGAAKNFKEALARYSKACELEN) | 205 | 240 | 3.90E-07 |
| [SEL1](http://smart.embl-heidelberg.de/smart/do_annotation.pl?DOMAIN=SEL1&TYPE=SMART&START=241&END=276&LENGTH=35&E_VALUE=3.3e-08&BLAST=GGCFNLGAMQYNGEGVTRNEKQAIENFKKGCKLGA) | 241 | 276 | 3.30E-08 |  | [SEL1](http://smart.embl-heidelberg.de/smart/do_annotation.pl?DOMAIN=SEL1&TYPE=SMART&START=241&END=276&LENGTH=35&E_VALUE=8.8e-07&BLAST=GGCFNLGAMQYNGEGATRNEKQAIENFKKGCKLGA) | 241 | 276 | 8.80E-07 |
|  |  |  |  |  |  |  |  |  |
| **Hp-1117** |  |  |  |  | **Jhp-1045** |  |  |  |
| **name** | **begin** | **end** | **E-value** |  | **name** | **begin** | **end** | **E-value** |
| [SEL1](http://smart.embl-heidelberg.de/smart/do_annotation.pl?DOMAIN=SEL1&TYPE=SMART&START=62&END=97&LENGTH=35&E_VALUE=1.9e-10&BLAST=LAYVLLGIMYENGRGVPKDEKKAAEYFQKAVDNDI) | 62 | 97 | 1.90E-10 |  | [SEL1](http://smart.embl-heidelberg.de/smart/do_annotation.pl?DOMAIN=SEL1&TYPE=SMART&START=62&END=97&LENGTH=35&E_VALUE=6.8e-11&BLAST=LAYILLGIMYENGRGVPKDYKKAVEYFQKAVDNDI) | 62 | 97 | 6.80E-11 |
| [SEL1](http://smart.embl-heidelberg.de/smart/do_annotation.pl?DOMAIN=SEL1&TYPE=SMART&START=98&END=133&LENGTH=35&E_VALUE=5.7e-10&BLAST=RGYNNLGVMYKEGRGVPKDEKKAVEYFRIATEKGY) | 98 | 133 | 5.70E-10 |  | [SEL1](http://smart.embl-heidelberg.de/smart/do_annotation.pl?DOMAIN=SEL1&TYPE=SMART&START=98&END=133&LENGTH=35&E_VALUE=4.1e-10&BLAST=RGYNNLGVMYKEGKGVPKDEKKAVEYFRIATEKGY) | 98 | 133 | 4.10E-10 |
| [SEL1](http://smart.embl-heidelberg.de/smart/do_annotation.pl?DOMAIN=SEL1&TYPE=SMART&START=134&END=169&LENGTH=35&E_VALUE=7.9e-08&BLAST=NAYINLGIMYMEGRGVPSNYVKATECFRKAMHKGN) | 134 | 169 | 7.90E-08 |  | [SEL1](http://smart.embl-heidelberg.de/smart/do_annotation.pl?DOMAIN=SEL1&TYPE=SMART&START=134&END=169&LENGTH=35&E_VALUE=2.5e-08&BLAST=NAYINLGIMYMEGRGVPSNYAKATECFRKAMHKGN) | 134 | 169 | 2.50E-08 |
| [SEL1](http://smart.embl-heidelberg.de/smart/do_annotation.pl?DOMAIN=SEL1&TYPE=SMART&START=170&END=208&LENGTH=38&E_VALUE=1.3e-07&BLAST=EAYILLGDIYYSGNDQLGIEPDKDKAIVYYKMAADMSS) | 170 | 208 | 1.30E-07 |  | [SEL1](http://smart.embl-heidelberg.de/smart/do_annotation.pl?DOMAIN=SEL1&TYPE=SMART&START=170&END=208&LENGTH=38&E_VALUE=3e-06&BLAST=EAYILLGDIYYSGNDQLGIEPDKDKAVVYYKMAADVSS) | 170 | 208 | 3.00E-06 |
| [SEL1](http://smart.embl-heidelberg.de/smart/do_annotation.pl?DOMAIN=SEL1&TYPE=SMART&START=209&END=244&LENGTH=35&E_VALUE=3e-05&BLAST=RAYEGLAESYQYGLGVEKDKKKAEEYMQKACDFDI) | 209 | 244 | 3.00E-05 |  | [SEL1](http://smart.embl-heidelberg.de/smart/do_annotation.pl?DOMAIN=SEL1&TYPE=SMART&START=209&END=244&LENGTH=35&E_VALUE=0.00024&BLAST=RAYEGLSESYRYGLGVEKDKKKAEEYMQKACDFDI) | 209 | 244 | 2.40E-04 |
|  |  |  |  |  |  |  |  |  |
| **Hp-0336** |  |  |  |  | **Jhp-1437** |  |  |  |
| **name** | **begin** | **end** | **E-value** |  | **name** | **begin** | **end** | **E-value** |
| [SEL1](http://smart.embl-heidelberg.de/smart/do_annotation.pl?DOMAIN=SEL1&TYPE=SMART&START=57&END=92&LENGTH=35&E_VALUE=8.4e-09&BLAST=NGCRFLGDFYENGKYVKKDLRKAAQYYSKACGLND) | 57 | 92 | 8.40E-09 |  | [low complexity](http://smart.embl-heidelberg.de/smart/show_segment.pl?NAME=SEG&START=13&END=24&BLAST=CLGALCLCGGL) | 13 | 24 | - |
| [SEL1](http://smart.embl-heidelberg.de/smart/do_annotation.pl?DOMAIN=SEL1&TYPE=SMART&START=93&END=128&LENGTH=35&E_VALUE=3.2e-05&BLAST=DGCLILGYKQYAGKGVVKNEKQAVKTFEKACRLGS) | 93 | 128 | 3.20E-05 |  | [SEL1](http://smart.embl-heidelberg.de/smart/do_annotation.pl?DOMAIN=SEL1&TYPE=SMART&START=105&END=136&LENGTH=31&E_VALUE=0.06&BLAST=EKCKDLAEFYFNVNDLKNALEYYSKSCKLNN) | 105 | 136 | 6.00E-02 |
|  |  |  |  |  | [SEL1](http://smart.embl-heidelberg.de/smart/do_annotation.pl?DOMAIN=SEL1&TYPE=SMART&START=137&END=172&LENGTH=35&E_VALUE=59&BLAST=EGCMLSATFYNDMIKGLKKDKKDLEYYSKACELNN) | 137 | 172 | 5.90E+01 |
|  |  |  |  |  | [SEL1](http://smart.embl-heidelberg.de/smart/do_annotation.pl?DOMAIN=SEL1&TYPE=SMART&START=173&END=208&LENGTH=35&E_VALUE=2.6e-07&BLAST=GGCSKLGGDYFFGEGVTKDFKKAFEYSAKACELND) | 173 | 208 | 2.60E-07 |
|  |  |  |  |  | [SEL1](http://smart.embl-heidelberg.de/smart/do_annotation.pl?DOMAIN=SEL1&TYPE=SMART&START=209&END=244&LENGTH=35&E_VALUE=1.1e-05&BLAST=KGCYALAAFYNEGKGVAKDEKQTTENLEKSCKLGL) | 209 | 244 | 1.10E-05 |
|  |  |  |  |  |  |  |  |  |
|  |  |  |  |  | **Jhp-0318** |  |  |  |
|  |  |  |  |  | **name** | **begin** | **end** | **E-value** |
|  |  |  |  |  | [SEL1](http://smart.embl-heidelberg.de/smart/do_annotation.pl?DOMAIN=SEL1&TYPE=SMART&START=27&END=62&LENGTH=35&E_VALUE=4.3e-06&BLAST=GGCGALGDLYDDGKGVEKNLIKAAQPYTKACELKE) | 27 | 62 | 4.30E-06 |
|  |  |  |  |  | [SEL1](http://smart.embl-heidelberg.de/smart/do_annotation.pl?DOMAIN=SEL1&TYPE=SMART&START=63&END=98&LENGTH=35&E_VALUE=0.00015&BLAST=LGCERLWSLYYYGRGVEKNLIKAAQYASKACDLNN) | 63 | 98 | 1.50E-04 |
|  |  |  |  |  | [SEL1](http://smart.embl-heidelberg.de/smart/do_annotation.pl?DOMAIN=SEL1&TYPE=SMART&START=99&END=134&LENGTH=35&E_VALUE=6.6e-10&BLAST=VGCKNLGFLYEYGEGVEKDLIKAAQYASKACDLNN) | 99 | 134 | 6.60E-10 |
|  |  |  |  |  | [SEL1](http://smart.embl-heidelberg.de/smart/do_annotation.pl?DOMAIN=SEL1&TYPE=SMART&START=135&END=170&LENGTH=35&E_VALUE=9.8e-05&BLAST=SGCDVLGFLYGSGKGVEKNLTKAAYFYSKACDLNE) | 135 | 170 | 9.80E-05 |
|  |  |  |  |  | [SEL1](http://smart.embl-heidelberg.de/smart/do_annotation.pl?DOMAIN=SEL1&TYPE=SMART&START=171&END=205&LENGTH=34&E_VALUE=0.00032&BLAST=LGCFNLGGLYNGQGVEKDLTKVAYLYSKACELKE) | 171 | 205 | 3.20E-04 |
|  |  |  |  |  | [SEL1](http://smart.embl-heidelberg.de/smart/do_annotation.pl?DOMAIN=SEL1&TYPE=SMART&START=206&END=241&LENGTH=35&E_VALUE=4.4e-07&BLAST=FGCGALAVLYINGQGVEKDLTKADQYISKACKLGD) | 206 | 241 | 4.40E-07 |
|  |  |  |  |  |  |  |  |  |
