# Supplementary material for: Helicobacter pylori Evolution: Lineage- Specific Adaptations in Homologs of Eukaryotic Sel1-Like Genes
Source: PLoS Comput Biol. 2007 Aug 10;3(8):e151. doi: 10.1371/journal.pcbi.0030151 (PMC1941758; doi:10.1371/journal.pcbi.0030151)
Supplement: Supplementary file 11 [file pcbi.0030151.st004.doc]

| **Table S4: d_N_ and d_S_ for each branch of *slr* gene-family phylogeny computed with FR Model** | | | | | | | | |
| --- | --- | --- | --- | --- | --- | --- | --- | --- |
| Branch | t | S | N | d_N_/d_S_ | d_N_ | d_S_ | S*d_S_ | N*d_N_ |
| 26..1 | 0.059 | 337.8 | 1021.2 | 0.2935 | 0.0123 | 0.0419 | 14.2 | 12.6 |
| 26..2 | 0.033 | 337.8 | 1021.2 | 0.138 | 0.0043 | 0.0311 | 10.5 | 4.4 |
| 26..27 | 0.065 | 337.8 | 1021.2 | 0.4364 | 0.0163 | 0.0374 | 12.6 | 16.7 |
| 27..3 | 0.143 | 337.8 | 1021.2 | 0.232 | 0.0261 | 0.1126 | 38 | 26.7 |
| 27..28 | 1.206 | 337.8 | 1021.2 | 0.4532 | 0.3092 | 0.6822 | 230.5 | 315.7 |
| 28..29 | 4.644 | 337.8 | 1021.2 | 0.0154 | 0.0916 | 5.951 | 2010.4 | 93.5 |
| 29..30 | 7.684 | 337.8 | 1021.2 | 0.0483 | 0.434 | 8.9919 | 3037.7 | 443.2 |
| 30..31 | 0.038 | 337.8 | 1021.2 | 0.1061 | 0.0041 | 0.0383 | 12.9 | 4.1 |
| 31..4 | 0.025 | 337.8 | 1021.2 | 0.1655 | 0.0038 | 0.0227 | 7.7 | 3.8 |
| 31..5 | 0.032 | 337.8 | 1021.2 | 0.12 | 0.0038 | 0.0316 | 10.7 | 3.9 |
| 30..6 | 0.137 | 337.8 | 1021.2 | 0.1515 | 0.0191 | 0.1264 | 42.7 | 19.6 |
| 29..32 | 1.894 | 337.8 | 1021.2 | 0.1182 | 0.2212 | 1.8709 | 632 | 225.9 |
| 32..33 | 0.3 | 337.8 | 1021.2 | 0.3181 | 0.0653 | 0.2052 | 69.3 | 66.7 |
| 33..34 | 0.268 | 337.8 | 1021.2 | 1.2546 | 0.0942 | 0.0751 | 25.4 | 96.2 |
| 34..35 | 0.029 | 337.8 | 1021.2 | 0.2684 | 0.0057 | 0.0212 | 7.2 | 5.8 |
| 35..13 | 0.046 | 337.8 | 1021.2 | 0.0717 | 0.0037 | 0.0509 | 17.2 | 3.7 |
| 35..14 | 0.036 | 337.8 | 1021.2 | 0.4321 | 0.0091 | 0.0211 | 7.1 | 9.3 |
| 34..15 | 0.096 | 337.8 | 1021.2 | 0.1386 | 0.0126 | 0.0912 | 30.8 | 12.9 |
| 33..36 | 0.596 | 337.8 | 1021.2 | 0.2308 | 0.1087 | 0.4712 | 159.2 | 111 |
| 36..37 | 0.082 | 337.8 | 1021.2 | 0.0001 | 0 | 0.1095 | 37 | 0 |
| 37..16 | 0.129 | 337.8 | 1021.2 | 0.3292 | 0.0286 | 0.087 | 29.4 | 29.3 |
| 37..17 | 0.053 | 337.8 | 1021.2 | 0.0793 | 0.0046 | 0.0576 | 19.5 | 4.7 |
| 36..18 | 0.011 | 337.8 | 1021.2 | 214.3419 | 0.0049 | 2.29E-05 | 0 | 5 |
| 32..38 | 0.036 | 337.8 | 1021.2 | 387.4244 | 0.0161 | 4.16E-05 | 0 | 16.4 |
| 38..39 | 0.803 | 337.8 | 1021.2 | 0.4403 | 0.2033 | 0.4618 | 156 | 207.6 |
| 39..19 | 0.228 | 337.8 | 1021.2 | 0.3187 | 0.0497 | 0.156 | 52.7 | 50.8 |
| 39..20 | 0.211 | 337.8 | 1021.2 | 1.0058 | 0.0705 | 0.0701 | 23.7 | 72 |
| 38..40 | 0.286 | 337.8 | 1021.2 | 999 | 0.1267 | 1.27E-04 | 0 | 129.4 |
| 40..21 | 1.326 | 337.8 | 1021.2 | 0.4781 | 0.3476 | 0.7271 | 245.6 | 355 |
| 40..22 | 0.974 | 337.8 | 1021.2 | 0.3284 | 0.2153 | 0.6558 | 221.5 | 219.9 |
| 28..41 | 0.47 | 337.8 | 1021.2 | 999 | 0.2086 | 2.09E-04 | 0.1 | 213 |
| 41..42 | 0.188 | 337.8 | 1021.2 | 999 | 0.0834 | 8.35E-05 | 0 | 85.1 |
| 42..43 | 5.052 | 337.8 | 1021.2 | 0.09 | 0.4794 | 5.3249 | 1798.9 | 489.5 |
| 43..44 | 0.024 | 337.8 | 1021.2 | 0.0274 | 0.0008 | 0.0297 | 10 | 0.8 |
| 44..7 | 0.082 | 337.8 | 1021.2 | 0.513 | 0.0221 | 0.0431 | 14.6 | 22.6 |
| 44..9 | 0.193 | 337.8 | 1021.2 | 0.1025 | 0.0203 | 0.1979 | 66.9 | 20.7 |
| 43..8 | 0.04 | 337.8 | 1021.2 | 0.0963 | 0.004 | 0.0418 | 14.1 | 4.1 |
| 42..45 | 3.71 | 337.8 | 1021.2 | 2.2449 | 1.4342 | 0.6389 | 215.8 | 1464.6 |
| 45..46 | 0.029 | 337.8 | 1021.2 | 0.012 | 0.0005 | 0.038 | 12.8 | 0.5 |
| 46..23 | 0.094 | 337.8 | 1021.2 | 0.2325 | 0.0172 | 0.0742 | 25.1 | 17.6 |
| 46..24 | 0.061 | 337.8 | 1021.2 | 0.1369 | 0.0079 | 0.058 | 19.6 | 8.1 |
| 45..25 | 0.062 | 337.8 | 1021.2 | 708.539 | 0.0273 | 0 | 0 | 27.9 |
| 41..47 | 15.733 | 337.8 | 1021.2 | 0.0321 | 0.6175 | 19.2308 | 6496.6 | 630.6 |
| 47..48 | 0.043 | 337.8 | 1021.2 | 0.0001 | 0 | 0.0575 | 19.4 | 0 |
| 48..10 | 0.048 | 337.8 | 1021.2 | 0.0326 | 0.0019 | 0.0587 | 19.8 | 2 |
| 48..11 | 0.106 | 337.8 | 1021.2 | 0.0147 | 0.002 | 0.1359 | 45.9 | 2 |
| 47..12 | 0.022 | 337.8 | 1021.2 | 0.2975 | 0.0047 | 0.0157 | 5.3 | 4.8 |
