# Supplementary material for: Helicobacter pylori Evolution: Lineage- Specific Adaptations in Homologs of Eukaryotic Sel1-Like Genes
Source: PLoS Comput Biol. 2007 Aug 10;3(8):e151. doi: 10.1371/journal.pcbi.0030151 (PMC1941758; doi:10.1371/journal.pcbi.0030151)
Supplement: Supplementary file 12 [file pcbi.0030151.st005.doc]

| **Table S5. Population differentiation at *hp0519* locus** | | | |  |  |  |
| --- | --- | --- | --- | --- | --- | --- |
| **Populations compared** | **π^†^_wSP(JC corrected)_** | **π^‡^_bSP(JC corrected)_** | **No. Fixed polymorphisms** | **No. Shared polymorphisms** | **F_ST_** | **PM^*^ test P value** |
| Europe vs Japan | 0.032vs 0.053 | 0.082 | 54 | 16 | 0.68 | <0.001 |
| Europe vs. Korea | 0.038 vs 0.044 | 0.051 | 5 | 21 | 0.45 | <0.001 |
| Europe vs Gambia | 0.035 vs 0.032 | 0.04 | 3 | 22 | 0.35 | <0.001 |
| Gambia vs. Korea | 0.032 vs 0.044 | 0.055 | 17 | 9 | 0.45 | <0.01 |
| Japan vs Korea | 0.053 vs 0.044 | 0.09 | 56 | 32 | 0.62 | <0.001 |
| Japan vs Korea^‡‡^ | 0.031 vs 0.049 | 0.06 | 5 | 15 | 0.51 | <0.001 |
| All Groups | NA | NA | NA | NA | 0.54 | <0.001 |
| †, Mean nucleotide diversity within subpopulations | | | | | | |
| ‡, Mean nucleotide diversity between subpopulations | | | | | | |
| *, Significance of observed F_ST_ was determined using a series of permutation-randomization tests | | | | | | |
| (K_s*_, K_st_, Z_s_, Z_s*_ (1) and S*_nn_* (2); all test statistics demonstrated identical significance levels. | | | | | | |
| ‡‡, based on 322 bp internal *hp0519* fragment; Japanese and Korean strains included here were | | | | | | |
| based on Figure 2A. | | | | | | |
| 1. Hudson RR et al, 1992. Mol. Biol. Evol. 9:138-151 | | | | | | |
| 2. Hudson RR. 2000. Genetics. 155:2011-2014 | | | | | | |
