# Supplementary material for: Helicobacter pylori Evolution: Lineage- Specific Adaptations in Homologs of Eukaryotic Sel1-Like Genes
Source: PLoS Comput Biol. 2007 Aug 10;3(8):e151. doi: 10.1371/journal.pcbi.0030151 (PMC1941758; doi:10.1371/journal.pcbi.0030151)
Supplement: Supplementary file 13 [file pcbi.0030151.st007.doc]

| **Table S7. Results for Hp0519 homology models for European and Japanese lineages.** | | | | | | | | |
| --- | --- | --- | --- | --- | --- | --- | --- | --- |
| Method | Program FUGUE | | | | Program LOOPP | | | |
| Query sequence | European | | Japan | | European | | Japan | |
| Rank | pdb-id | score | pdb-id | score | pdb-id | score | pdb-id | score |
| 1 | 1klx | 23.6 | 1klx | 31.2 | 1ouv | 3.19 | 1ouv | 3.12 |
| 2 | 1ya0 | 10.8 | 1ya0 | 8.8 | 1klx | 2.17 | 1klx | 2.77 |
| 3 | 1w3b | 9.1 | 1fch | 6.7 | 1fch | 1.75 | 1elr | 1.86 |
| 4 | 1ool | 8.1 | 1w3b | 6 | 1na0 | 1.64 | 1na0 | 1.75 |
| 5 | 1fch | 8.1 | 1ft1 | 4.9 | 1qoy | 1.55 | 1fch | 1.66 |
| 6 | 1e96 | 6.4 | 1ool | 4.7 | 1qqe | 1.46 | 1qqy | 1.56 |
| SLR proteins: 1klx, 1ouv | | | | | | | | |
| TPR proteins: 1ya0, 1w3b, 1ool, 1fch, 1e96, 1na0, 1qqe, 1qqy | | | | | | | | |
