# Supplementary material for: Helicobacter pylori Evolution: Lineage- Specific Adaptations in Homologs of Eukaryotic Sel1-Like Genes
Source: PLoS Comput Biol. 2007 Aug 10;3(8):e151. doi: 10.1371/journal.pcbi.0030151 (PMC1941758; doi:10.1371/journal.pcbi.0030151)
Supplement: Supplementary file 14 [file pcbi.0030151.st008.doc]

| **Table S8. ML parameter estimates of selection pressures acting on *H. pylori Hp1117* SLR protein.** | | | | | | | | | |
| --- | --- | --- | --- | --- | --- | --- | --- | --- | --- |
| **Model Code** | **InL** | **Tree Length** | **κappa** | **d_N_/d_S_** | **Estimates of Parameters** | **LRT [d.f.]** | **χ^2^** | **P** | **Positively selected sites** |
| M0 (1-ratio) | -3297.27 | 1.742 | 5.029 | 0.131 | ω=0.131 | - | - | - | Not Applicable |
| M1(neutral) | -3202.14 | 1.808 | 5.598 | 0.196 | p0 = 0.804, ω0 = 0.0; p1 = 0.195, ω1 = 1 | - | - | - | Not Applicable |
| M2 (selection) | -3202.14 | 1.808 | 5.6 | 0.195 | p0 = 0.804, ω0 = 0.0; p1 = 0.195, ω1 = 1; (p2) = 0 | M0 vs M2 [2] M1 vs M2 [2] | 190.266 0.0 | <0.000 N.S. |  |
| M3 (discrete) | -3186.36 | 1.915 | 5.302 | 0.179 | p0 = 0.768, ω0 = 0.0; p1 = 0.179, ω1 = 0.386; p2 = 0.052, **ω2 = 2.107** | M0 vs M3 [4] M1 vs M3 [4] M2 vs M3 [2] | 221.826 31.56 31.56 | <0.000 <0.000 <0.000 | ***3Y***, **8A**, **21T** ***29Q***, **64S**, 84Y, ***131T***, ***157A***, **162C**, **208**I, ***218S***, **222Q**, **232K** |
| M7 (β) | -3191.75 | 1.812 | 4.876 | 0.131 | *p* = 0.034, *q* = 0.226 |  |  |  |  |
| M8 (β & ω) | -3187.37 | 1.902 | 5.268 | 0.176 | *p* = 0.079, *q* = 0.914; p0 = 0.95, (p1) = 0.0495, **(ω2) = 2.122** | M7 vs M8 [2] | 8.756 | <0.01 | ***3Y***, **8A**, **21T**, ***29Q***, **64S**, 84Y, 131T, 157A, **162C**, **208**I, 218S, **222Q**, **232K** |
| FR | -3252.54 | 1.739 | 5.034 | NA | Individual ω for each branch | FR vs M0 [63] | 89.464 | <0.01 | **Positive selection acts on certain lineages in the tree** |
| Please see footnotes to table S6a for explanation | | | | | | | | | |
