# Supplementary material for: Helicobacter pylori Evolution: Lineage- Specific Adaptations in Homologs of Eukaryotic Sel1-Like Genes
Source: PLoS Comput Biol. 2007 Aug 10;3(8):e151. doi: 10.1371/journal.pcbi.0030151 (PMC1941758; doi:10.1371/journal.pcbi.0030151)
Supplement: Supplementary file 15 [file pcbi.0030151.st009.doc]

| **Table S9. ML parameter estimates of selection pressures acting on *H. pylori Hp0211* SLR protein.** | | | | | | | | | |
| --- | --- | --- | --- | --- | --- | --- | --- | --- | --- |
| **Model Code** | **InL** | **Tree Length** | **κ** | **d_N_/d_S_** | **Estimates of Parameters** | **LRT [d.f.]** | **χ^2^** | **P** | **Positively selected sites** |
| M0 (1-ratio) | -3029.255 | 1.504 | 4.86 | 0.222 | ω=0.222 | - | - | - | Not Applicable |
| M1(neutral) | -2975.285 | 1.553 | 5.21 | 0.306 | p0 = 0.693, ω0 = 0.0; p1 = 0.306, ω1 = 1 | - | - | - | Not Applicable |
| M2 (selection) | -2970.721 | 1.592 | 5.501 | 0.37 | p0 = 0.690, ω0 = 0.0; p1 = 0.289, ω1 = 1; (p2) = 0.019, **(ω2) = 4.078** | M0 vs M2 [2] M1 vs M2 [2] | 117.068 9.128 | <0.000 <0.01 | 17T, **40A**, **50T**, 60N, 235S |
| M3 (discrete) | -2960.531 | 1.614 | 4.967 | 0.265 | p0 = 0.624, ω0 = 0.0; p1 = 0.333, ω1 = 0.460; p2 = 0.042, **ω2 = 2.631** | M0 vs M3 [4] M1 vs M3 [4] M2 vs M3 [2] | 137.448 29.508 20.38 | <0.000 <0.000 <0.000 | **17T**, **40A**, **50T**, **60N**, 143H, 150P, **235S** |
| M7 (β) | -2967.771 | 1.575 | 4.122 | 0.221 | *p* = 0.104, *q* = 0.336 |  |  |  |  |
| M8 (β & ω) | -2961.196 | 1.615 | 4.989 | 0.268 | *p* = 0.164, *q* = 0.756; p0 = 0.966, (p1) = 0.033,**(ω) = 2.857** | M7 vs M8 [2] | 13.15 | <0.01 | **17T**, **40A**, **50T**, **60N**, 150P, **235S** |
| FR | -2981.87 | 1.995 | 4.876 | NA | Independent ω for each branch | FR vs M0 [65] | 94.77 | <0.01 | **Positive selection acts on certain lineages in the tree** |
| Please see footnotes to table S6a for explanation | | | | | | | | | |
