# Supplementary material for: Helicobacter pylori Evolution: Lineage- Specific Adaptations in Homologs of Eukaryotic Sel1-Like Genes
Source: PLoS Comput Biol. 2007 Aug 10;3(8):e151. doi: 10.1371/journal.pcbi.0030151 (PMC1941758; doi:10.1371/journal.pcbi.0030151)
Supplement: Supplementary file 16 [file pcbi.0030151.st010.doc]

| **Table S10. ML parameter estimates of selection pressures acting on *H. pylori* Hp0160 SLR protein.** | | | | | | | | | |
| --- | --- | --- | --- | --- | --- | --- | --- | --- | --- |
| **Model Code** | **InL** | **Tree Length** | **κ** | **d_N_/d_S_** | **Estimates of Parameters** | **LRT [d.f.]** | **χ^2^** | **P** | **Positively selected sites** |
| M0 (1-ratio) | -5110.636 | 2.503 | 6.999 | 0.257 | ω=0.257 | - | - | - |  |
| M1(neutral) | -4954.94 | 2.541 | 7.717 | 0.352 | p0 = 0.647, ω0 = 0.0; p1 = 0.352, ω1 = 1 | - | - | - |  |
| M2 (selection) | -4926.533 | 2.689 | 8.137 | 0.444 | p0 = 0.645, ω0 = 0.0; p1 = 0.338, ω1 = 1; (p2) = 0.016, **(ω2) = 6.439** | M0 vs M2 [2] M1 vs M2 [2] | 368.21 56.814 | <0.000 <0.000 | **97I, 146A, 174V, 265L,** 305M |
| M3 (discrete) | -4899.269 | 2.725 | 7.544 | 0.335 | p0 = 0.749, ω0 = 0.034; p1 = 0.234, ω1 = 0.943; p2 = 0.016, **ω2 = 5.509** | M0 vs M3 [4] M1 vs M3 [4] M2 vs M3 [2] | 422.73 111.34 54.528 | <0.000 <0.000 <0.000 | **97I, 146A, 174V, 265L** |
| M7 (β) | -4927.009 | 2.615 | 6.915 | 0.246 | *p* = 0.097, *q* = 0.299 |  |  |  |  |
| M8 (β & ω) | -4901.64 | 2.742 | 7.373 | 0.321 | *p* = 0.105, *q* = 0.339; p0 = 0.982, (p1) = 0.017, **(ω) = 5.181** | M7 vs M8 [2] | 50.74 | <0.000 | **97I, 146A, 174V, 265L,** 305M |
| FR | -5068.934 | 2.52 | 7.047 | NA | Independent ω for each branch | FR vs M0 [63] | 83.04 | <0.05 | **Positive selection acts on certain lineages in the tree** |
| Please see footnotes to table S6a for explanation | | | | | | | | | |
