# Supplementary material for: Helicobacter pylori Evolution: Lineage- Specific Adaptations in Homologs of Eukaryotic Sel1-Like Genes
Source: PLoS Comput Biol. 2007 Aug 10;3(8):e151. doi: 10.1371/journal.pcbi.0030151 (PMC1941758; doi:10.1371/journal.pcbi.0030151)
Supplement: Supplementary file 17 [file pcbi.0030151.st011.doc]

| **Table S11. ML parameter estimates of selection pressures acting on *H. pylori Hp0235* SLR protein.** | | | | | | | | | |
| --- | --- | --- | --- | --- | --- | --- | --- | --- | --- |
| **Model Code** | **InL** | **Tree Length** | **κ** | **d_N_/d_S_** | **Estimates of Parameters** | **LRT [d.f.]** | **χ^2^** | **P** | **Positively selected sites** |
| M0 (1-ratio) | -4289.552 | 1.614 | 4.137 | 0.189 | ω=0.189 | - | - | - | Not Applicable |
| M1(neutral) | -4213.072 | 1.632 | 4.59 | 0.267 | p0 = 0.732, ω0 = 0.0; p1 = 0.267, ω1 = 1 | - | - | - | Not Applicable |
| M2 (selection) | -4205.343 | 1.693 | 4.851 | 0.33 | p0 = 0.730, ω0 = 0.0; p1 = 0.253, ω1 = 1; (p2) = 0.016, **(ω2) = 4.747** | M0 vs M2 [2] M1 vs M2 [2] | 168.42 15.458 | <0.000 <0.000 | 3V, **17S,** 26Q, **47T,** 57G, ***88T*** |
| M3 (discrete) | -4177.986 | 1.721 | 4.226 | 0.229 | p0 = 0.887, ω0 = 0.057; p1 = 0.097, **ω1 = 1.246**; p2 = 0.014, **ω2 = 3.851** | M0 vs M3 [4] M1 vs M3 [4] M2 vs M3 [2] | 223.13 70.172 54.714 | <0.000 <0.000 <0.000 | **2G**, **3V**, 5F, ***8I***, ***9L***, **11C**, **14F**, **17S**, **19N, 20A, 26Q,** 33V, **36K, 47T**, ***48S***, **57G**, ***76R***, **88T**, **105Y**, **111T, 112T, 120D,** 121A, **150D, 170V, 173V, 200D, *207A,* 235G, 264N, 290A,** *292P*, **332M** |
| M7 (β) | -4194.212 | 1.675 | 3.945 | 0.178 | *p* = 0.102, *q* = 0.471 |  |  |  |  |
| M8 (β & ω) | -4180.001 | 1.691 | 4.219 | 0.219 | *p* = 1.081, *q* = 14.33; p0 = 0.907, (p1) = 0.093, **(ω) = 1.687** | M7 vs M8 [2] | 28.422 | <0.000 | **2G**, **3V**, 8I, ***9L***, **11C**, **14F**, **17S**, **19N**, **20A**, **26Q**, **36K**, **47T**, 48S, **57G**, 76R, **88T**, ***105Y***, **111T**, **112T**, **120D**, 150D, ***170V***, **173V**, ***200D***, 207A, **235G**, **264N**, **290A**, ***292P***, ***332M*** |
| FR | -4256.601 | 1.613 | 4.131 | NA | Independent ω for each branch | FR vs M0 [63] | 65.902 | N.S. | Gene evolves rapidly but rates do not vary among lineages |
| Please see footnotes to table S6a for explanation | | | | | | | | | |
