# Supplementary material for: Helicobacter pylori Evolution: Lineage- Specific Adaptations in Homologs of Eukaryotic Sel1-Like Genes
Source: PLoS Comput Biol. 2007 Aug 10;3(8):e151. doi: 10.1371/journal.pcbi.0030151 (PMC1941758; doi:10.1371/journal.pcbi.0030151)
Supplement: Supplementary file 18 [file pcbi.0030151.st012.doc]

| **Table S12. ML parameter estimates of selection pressures acting on *H. pylori Hp0628* SLR protein.** | | | | | | | | | |
| --- | --- | --- | --- | --- | --- | --- | --- | --- | --- |
| **Model Code** | **InL** | **Tree Length** | **κ** | **d_N_/d_S_** | **Estimates of Parameters** | **LRT [d.f.]** | **χ^2^** | **P** | **Positively selected sites** |
| M0 (1-ratio) | -4428.268 | 1.875 | 4.112 | 0.063 | ω=0.063 | - | - | - | Not Applicable |
| M1(neutral) | -4383.485 | 1.916 | 4.974 | 0.143 | p0 = 0.856, ω0 = 0.0; p1 = 0.143, ω1 = 1 | - | - | - | Not Applicable |
| M2 (selection) | -4360.269 | 1.93 | 4.2 | 0.078 | p0 = 0.815, ω0 = 0.0; p1 = 0.034, ω1 = 1; (p2) = 0.149, (ω2) = 0.289 | M0 vs M2 [2] M1 vs M2 [2] | 136 46.432 | <0.000 <0.000 | **None** |
| M3 (discrete) | -4360.185 | 1.922 | 4.176 | 0.076 | p0 = 0.855, ω0 = 0.004; p1 = 0.111, ω1 = 0.388; p2 = 0.033, ω2 = 0.881 | M0 vs M3 [4] M1 vs M3 [4] M2 vs M3 [2] | 136.17 46.6 0.168 | <0.000 <0.000 **N.S.** | **None** |
| M7 (β) | -4360.169 | 1.922 | 4.17 | 0.075 | *p* = 0.060, *q* = 0.710 |  |  |  |  |
| M8 (β & ω) | -4360.458 | 3.265 | 4.161 | 0.289 | *p* = 0.060, *q* = 0.713; p0 = 0.999, (p1) = 0.0008,(ω) = ι | M7 vs M8 [2] | -0.578 | **N.S.** | **None** |
| FR | -4391.253 | 1.88 | 4.119 | NA | Independent ω for each branch | FR vs M0 [63] | 74.03 | **N.S. (>.1)** | **Gene evolution constrained by strong purifying selection** |
| Please see footnotes to table S6a for explanation | | | | | | | | | |
