# Supplementary material for: Helicobacter pylori Evolution: Lineage- Specific Adaptations in Homologs of Eukaryotic Sel1-Like Genes
Source: PLoS Comput Biol. 2007 Aug 10;3(8):e151. doi: 10.1371/journal.pcbi.0030151 (PMC1941758; doi:10.1371/journal.pcbi.0030151)
Supplement: Supplementary file 19 [file pcbi.0030151.st013.doc]

| **Table S13. Primers used in this study.** | | | |  |
| --- | --- | --- | --- | --- |
| **Name** | **Sequence** | | | **Remarks** |
| *hp0519R1* | 5’-GTTGCCGTTTTCRCTTTGTATAGCT | | | 24 bp deletion in *hp0519* |
| *Luni1* | 5’-ACATTTTGGCTAAATAAACGCTG | | |  |
| *hp0519R2* | 5’-CGATTCATTGATCTTTCAGGTTTGAG | | | 15 bp deletion in *hp0519* |
| *hp0519F1* | 5’-GGCTTTTTCATAGCCAAATTCTGCG | | |  |
| *hp0519/520A4F* | 5’-ATTATAGCAAAAAATATAAAACCAT | | | *hp0519* amplification and sequencing |
| *Lj1R* | 5’-TTGGTTTGGCTAGAAAAACAAGCT | | |  |
| *hp0519A3R* | 5’-ATTAGAAAACATGCGAACCACA | | |  |
| *hp0519A2F* | 5’-GGGCGTGAAAGTGGATTACAAAAAA | | |  |
| *hp0518A1F* | 5’-CAAGTGTCTATTATAATGGAAAAATA | | |  |
| *hp0517F1* | 5’-GTGGCCGAAAAAAAGGTTTTTTTAAA | | | *hp0518* amplification and sequencing |
| *hp0519R4* | 5’-TGGGATCGCTTATAAAGAAGGGCTT | | |  |
| *hp0519F2* | 5’-GCTAATATATTCATCAGCTATACAAA | | | *hp0520* amplification and sequencing |
| *hp0522R* | 5’-ACAGCGGTTGCTAGTTTTCTAAACA | | |  |
| *hp0159F* | 5'-TGACCATTTTAGAAAAGCGAGTCTT | | | *hp0160* amplification and sequencing |
| *hp0162R* | 5'-CGATGATGATGTGGTCGCGCTTT | | |  |
| *hp0160F* | 5'-TGGCGATGGCGTGGATCAGA | | |  |
| *hp0160R* | 5'-GCGTGTCGTTTTGCGCGTCAT | | |  |
| *hp0210F* | 5'-ATCTGGTTGCTTTATGATGGG | | | *hp0211* amplification and sequencing |
| *hp0212R* | 5'-GCACGAGCGATGCGCGATTT | | |  |
| *hp0211F* | 5'-CCGCTTGCTTTTCGTCCTTTGC | | |  |
| *hp0211R* | 5'-TGGGGTCTTGTGTTTGGGC | | |  |
| *hp0234F* | 5'-TGAGCGCGTTTTTAATGGTGGCTTT | | | *hp0235* amplification and sequencing |
| *hp0236R* | 5'-GCCATGGCATTAAAGGCGAACAA | | |  |
| *hp0235F* | 5'-GCCCGCTAGAGCGAAGCAACC | | |  |
| *hp0235R* | 5'-ACCGCATGCCAGGCTGATGA | | |  |
| *hp0626F* | 5'-TGGCGTGCATTTTCGTTCCA | | | *hp0627* amplification and sequencing |
| *hp0629R* | 5'-TGCTGCCCGTCAATGATGACA | | |  |
| *hp0627F* | 5'-AATGCGGCCGGGTGTTTTG | | |  |
| *hp0627R* | 5'-CCCCATCGCATGCCATTTG | | |  |
| *hp1116F* | 5'-GCTCCTGCGGTGGCTTCGTT |  |  | *hp1117* amplification and sequencing |
| *hp1118R* | 5'-GGCATGCCCGCTGATGTGAA | | |  |
| *hp1117F* | 5'-TCTTTTTCCACGCCTAACCCATAC | | |  |
| *hp1117R* | 5'-CTTGGTGCAGAACACCTTGA | | |  |
